# Diffusion and Surface Effects on Sodium‐Promoted MoS_2_ Growth Observed in *Operando*

**DOI:** 10.1002/smtd.202500813

**Published:** 2025-08-07

**Authors:** Jehyun Oh, Yoonbeen Kang, Jae Hun Seol, Yong Hui Kim, Jinyoung Seo, Sang Uck Lee, Sang‐Yong Ju

**Affiliations:** ^1^ Department of Chemistry Yonsei University Seodaemun‐Gu Seoul 03722 Republic of Korea; ^2^ School of Chemical Engineering Sungkyunkwan University Suwon 16419 Republic of Korea

**Keywords:** diffusion, growth kinetics, molybdenum disulfide, real‐time, substrate

## Abstract

Understanding precursor diffusion and substrate interaction is key to advancing chemical vapor deposition (CVD) of transition metal dichalcogenides (TMCs), yet direct observation has remained a challenge due to limited real‐time observation. Here, MoS_2_ growth is directly monitored to investigate the kinetics and influence of the precursor/sodium droplet eutectic (SODE). Serving as a catalyst, SODE migrates from the basal plane to the edges and substrate interface, promoting growth and enabling grain translation and rotation. Kinetic analysis shows MoS_2_ grows more readily on its own surface than on SiO_2_, indicating a thermodynamic‐kinetic interplay supported by density functional theory calculations. Notably, larger SODE droplets enhance such grain dynamics, while submicron‐scale SODE exhibits extended diffusion, enabling uniform, large‐area growth. These findings highlight the critical role of molten metal diffusion in growth continuity and provide new insights for optimizing scalable, cost‐effective TMC fabrication.

## Graphical abstract

Understanding precursor diffusion and substrate interaction is key to advancing chemical vapor deposition (CVD) of transition metal dichalcogenides (TMCs), yet direct observation has remained a challenge. The diffusion and surface effects of MoS_2_ growth are investigated by the amounts of sodium in real‐time.

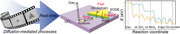

## 1 Introduction

During the chemical vapor deposition (CVD) growth of transition metal dichalcogenides (TMCs), precursor diffusion and substrate‐mediated surface pressure play a critical role in achieving high‐quality, large‐area films^[^ ^1^, ^2^, ^3^ ^]^ for high‐end applications. These factors significantly influence the structural characteristics of TMCs, including their shape,^[^ ^4^, ^5^, ^6^, ^7^, ^8^, ^9^ ^]^ edges,^[^ ^4^, ^8^, ^9^, ^10^, ^11^, ^12^ ^]^ and oriented epitaxy.^[^ ^13^, ^14^, ^15^, ^16^, ^17^ ^]^ Molybdenum disulfide (MoS_2_), a widely studied TMC, is synthesized in a CVD chamber at high temperatures using molybdenum (Mo) and sulfur (S) derivatives as precursors. These precursors diffuse—whether in gaseous, adatom, or flake form—before chemically bonding to the nucleus on the substrate.^[^ ^6^, ^7^, ^18^ ^]^ Despite several theoretical predictions,^[^ ^6^, ^7^, ^8^, ^19^ ^]^ the experimental understanding of diffusion mechanisms during growth, such as adsorption energy and activation energy for diffusion, remains incomplete. Gaining deeper insights into these processes is essential for fully comprehending and optimizing CVD‐based growth.

Especially, although the interaction between MoS_2_ and the substrate significantly influences its quality, the interaction with silicon dioxide (SiO_2_), one of the most commonly used substrates for CVD growth,^[^ ^3^, ^5^, ^20^, ^21^, ^22^ ^]^ is not well understood. The diffusion behavior of individual precursors on SiO_2_ plays a crucial role in controlling the growth process. When MoS_2_ growth is diffusion‐limited, it tends to form dendritic structures,^[^ ^6^ ^]^ whereas reduced diffusion typically results in various polygonal shapes, such as triangles, hexagons, and truncated forms.^[^ ^4^, ^5^, ^9^, ^21^ ^]^ This variation in growth dynamics leads to randomly oriented grains, ultimately producing a polycrystalline film. Understanding the substrate interaction is beneficial for the controlled growth of CVD.

In contrast, the epitaxial interaction between MoS_2_ and a lattice‐matching substrate enables the growth of well‐aligned grains.^[^ ^13^, ^14^, ^15^, ^16^, ^17^, ^23^ ^]^ For instance, leveraging the binding affinity between a gold surface with a specific crystallographic orientation and MoS_2_ sulfides has been shown to produce large‐area MoS_2_ with single‐crystal orientation,^[^ ^13^ ^]^ where triangular grains grow in the same direction and seamlessly merge. Similarly, the close lattice match between MoS_2_ and sapphire substrates, such as miscut *c*‐plane sapphire, facilitates epitaxial growth.^[^ ^14^, ^15^, ^16^ ^]^ Additionally, mica has also been reported to support epitaxial MoS_2_ growth.^[^ ^17^ ^]^ Minimizing grain mismatch—caused by factors such as translation and rotation, aside from substrate morphology—is expected to enhance the quality of MoS_2_ films.^[^ ^16^ ^]^

Recently, alkali metal halide molten salts^[^ ^1^, ^3^, ^24^, ^25^, ^26^, ^27^, ^28^ ^]^ have garnered attention as catalysts for the rapid and reproducible CVD growth of various TMCs. For instance, Li et al.^[^ ^24^ ^]^ demonstrated that sodium chloride acts as a catalyst in MoS_2_ nanoribbon growth via the vapor‐liquid‐solid (VLS) mechanism, emphasizing the crucial role of alkali metals. Additionally, our group,^[^ ^21^ ^]^ along with others,^[^ ^29^, ^30^ ^]^ has shown that alkali metals facilitate TMC growth, particularly along the edges. More recently, Jiang and colleagues^[^ ^31^ ^]^ demonstrated that continuous, large‐area single‐crystalline MoS_2_ can be achieved using sodium embedded in molten glass. As a result, the diffusional behavior and substrate interaction of fluidized sodium have emerged as critical factors. However, despite their catalytic effectiveness, the diffusive behavior of these alkali metals—an essential factor for achieving large‐area growth—remains largely unexplored.

*Operando* CVD growth, which enables real‐time observation and control of MoS_2_ formation,^[^ ^21^, ^28^, ^32^, ^33^, ^34^ ^]^ is expected to provide insights into both the growth trajectory and diffusion behavior. For example, Oh et al.^[^ ^21^ ^]^ reported that the shapes and edges of MoS_2_ grains are influenced by the partial pressures of individual precursors catalyzed by a sodium droplet eutectic (SODE), with growth occurring primarily along the edges. Therefore, real‐time monitoring of diffusion characteristics dependent on both precursors and the substrate could significantly enhance our understanding of diffusion‐driven surface reactions and their impact on overall growth dynamics.^[^ ^6^, ^7^ ^]^

In this study, we explored the diffusion‐ and substrate‐dependent growth behavior of MoS_2_ in relation to precursor/SODE under *operando* conditions. Using an integrated CVD microscope (ICVDM), we captured real‐time growth events, enabling the analysis of diffusion dynamics and substrate effects. This approach allowed us to elucidate diffusional pathways and the preferential positioning of SODE. Additionally, we observed distinct growth kinetics of MoS_2_ on MoS_2_ and SiO_2_ basal planes are observed, which led to the determination of activation energy for precursor diffusion. To further understand these kinetic and underlying thermodynamic mechanisms, we performed density functional theory (DFT) calculations to evaluate the adsorption energy and activation energy for precursor diffusion on MoS_2_ and SiO_2_. Finally, we observed the role of SODE for the formation of a uniform film compared to the growth without SODE.

## 2 Results and Discussion

### 2.1 Diffusional Pathway of Precursor/SODE in MoS_2_ Growth

**Figure 1:**
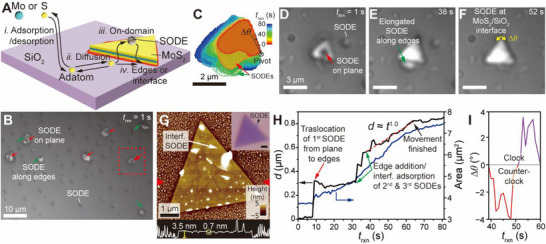
Diffusional pathway of precursor/SODE during MoS_2_ growth. A) Schematic illustrating SODE‐promoted bulk triangular MoS_2_ growth on a SiO_2_ surface. B) Large‐area optical image of ML grains containing a large number of SODEs on substrate, on MoS_2_ basal plane, and at edges of grains. C) Growth contour map of the grain indicated by the dashed box in (B), derived from overlaid MoS_2_/SODE edge images taken at 3‐s intervals. The bulged regions result from either edge translocation or adsorption of SODE. D–F) Time‐lapse optical images showing bulk MoS_2_ grain growth with translocating SODE: (D) SODE on the grain basal plane, (E) SODE translocating to the edges, and (F) translational and rotational movements of the grain floating on interfacial SODE. Scale bar: 3 µm in (D–F). G) AFM image of bulk MoS_2_ with interfacial SODE, with the bottom panel showing the height profile along the triangles in (G), revealing a 3.5 nm‐thick (or five‐layered) grain with an interfacial SODE height of ≈0.7 nm. Inset: optical image showing no extra contrast in the basal plane. H) Analysis of translational motion (left) and area change (right) of the grain in (C–F) as a function of time. I) Plot of Δ*θ vs*. *t*_rxn_, centered on the pivot in (C).

**Figure** **1**A presents a schematic of the SODE‐promoted MoS_2_ crystal growth process during the CVD process.^[^ ^21^ ^]^ The growth of solid crystals begins with the adsorption of precursor gases onto the substrate (step *i*). The precursor adatoms then diffuse across the substrate (step *ii*) and undergo a translocation process to reach a nucleus or growing flake (step *iii*). Finally, some of these adatoms incorporate into the most energetically favorable sites on the edges (step *iv*), explained by the terrace‐ledge‐kink (TLK) model^[^ ^35^ ^]^ and transition state theory.^[^ ^4^ ^]^ Notably, the edges of the flake are energetically more favorable than both the flake's surface and the substrate, allowing the precursors to remain and react at the crystal's binding sites, completing the binding of a precursor atom.^[^ ^7^ ^]^ These sequential processes influence the growth rates, which were analyzed using ICVDM.

Real‐time observations revealed that precursor incorporation into the grain follows growth pathways from the basal plane to the edges or interface. (Video S1, Supporting Information) shows a movie clip where SODE, initially positioned on the domain, suddenly migrates to the edges. The video shows that four among nine MoS_2_ grains have SODE on the basal plane. SODE forms in situ through the decomposition of coated molybedenum trioxide (MoO_3_)/sodium cholate (SC) during the temperature ramping process. It becomes supersaturated with Mo and S during the induction phase, after which it deposits nanometer‐scale, supersaturated MoS_2_ layers along the edges, promoting MoS_2_ growth.^[^ ^21^ ^]^ Figure 1B displays the optical image that multi‐layer (ML) grains and 1 µm‐sized SODE exist over a substrate at *t*_rxn_ = 1 s. As indicated by color‐coded arrows, some SODEs are placed on the MoS_2_ basal plane, whereas other SODEs are either at the edges or substrate. Figure 1C displays the growth contour map of triangular MoS_2_, created by overlaying color‐coded outlines from Video S1 (Supporting Information).

The map reveals significant translation and rotation of the flake during the reaction. Figure 1D–F present optical images showing the reaction time (*t*_rxn_)‐dependent triangular growth facilitated by SODE. Initially, SODEs are distributed across the thick triangular MoS_2_ and the entire SiO_2_/Si surfaces (Figure 1D). After *t*_rxn_ = 38 s, most of the SODEs on the substrate vanish, while those on the domain migrate toward the edges, and additional SODEs adsorb onto the edges, forming elongated structures (Figure 1E). As the reaction progresses, SODE gradually infiltrates the interface between MoS_2_ and the substrate due to capillary action (Figure 1F). This behavior indicates that SODE interacts more strongly with the edges than with the basal plane. During the growth on the interfacial SODE, the triangular flake undergoes both translation and rotation. Once all the SODE disappears, the MoS_2_ grain shows reduced growth rates and continues to exhibit both translational and rotational movements.

The movement of SODE from the basal plane to the edges follows the conventional surface growth process depicted in Figure 1A. The distinct SODE morphologies observed on the basal plane (spherical) and at the edges (ellipsoidal) suggest a strong adhesion energy between SODE and MoS_2_ edges. This aligns well with the scooting growth mechanism of SODE along the edges,^[^ ^21^ ^]^ as well as the salt‐assisted growth mechanism.^[^ ^24^, ^26^ ^]^ Furthermore, capillary action between two planes—previously observed in graphene intercalation by water^[^ ^36^, ^37^ ^]^—facilitates the interfacial adsorption of SODE. Given the densities of bulk MoS_2_, molten sodium, and SODE (5.06 and 0.93 g cm^−3^, and an intermediate value between them, respectively), MoS_2_ would typically be expected to maintain direct contact with the substrate. However, the reversal of this stacking order suggests that SODE exhibits stronger binding interactions with both SiO_2_ and MoS_2_. This is further confirmed by atomic force microscopy (AFM) measurements (Figure 1G), which reveal a 3.5 nm‐thick (or five‐layered) grain with a 0.7 nm‐thick interfacial SODE layer (white arrow), along with a few nanometer‐tall SODE particles on the substrate.^[^ ^26^ ^]^ The presence of SODE beneath the MoS_2_, rather than as an additional MoS_2_ layer, is supported by the optical image (inset), which shows no extra contrast. Additionally, the faint contrast of the few‐nanometer‐tall SODE structures at the edges (arrow) further supports this interpretation. Moreover, our recent work^[^ ^38^ ^]^ and other work^[^ ^31^, ^39^ ^]^ confirm the existence of underlying sodium beneath the grains.

Figure 1H depicts the time‐dependent translational movement and area expansion of the MoS_2_ as shown in Figure 1C–F. Although the translational movement approaches the optical resolution limit, its coupling with rotational motion enables distinction of the movement. Following multiple SODE‐related events, including translocation and edge addition, both the translational motion (left axis) and area increase (right axis) show an approximately linear correlation with *t*_rxn_. The 1D translational movement distance *d via* grain diffusion is described by Equation (1):^[^ ^40^ ^]^

(1) $$d={\left(Dt\right)}^{b}$$

where *D* is the diffusivity of the flake in µm/s, *t* is time in seconds, and *b* is an exponent that characterizes the nature of diffusion—0.5 indicating random diffusion and 1.0 representing directional diffusion.^[^ ^28^, ^40^ ^]^ The translational region (red dashed line) fitted to this equation yields *D* = 0.009 µm s^−^ and *b* ≈ 1.0, suggesting that the translational motion of the MoS_2_ flake is directional. Similar behaviors are observed for the different grains as well (Figure S1A–D, Supporting Information), showing the average *b* ≈ 0.98. This behavior is most likely driven by the influence of the argon carrier gas flow containing sulfur vapor.

Figure 1I shows the rotational angle difference (Δ*θ*) of the overlaid triangular MoS_2_ silhouettes, aligned to the pivot (Figure 1C), as a function of *t*_rxn_. A slight rotation is observed when SODE is introduced and adsorbed at the edges (Figure 1E). The maximum rotations reach ±4 degrees, alternating between counterclockwise and clockwise directions. This rotational behavior suggests weak adhesion between the MoS_2_ flake and the substrate, allowing for rotational freedom during growth.

### 2.2 Substrate‐Dependent Growth Kinetics

**Figure 2:**
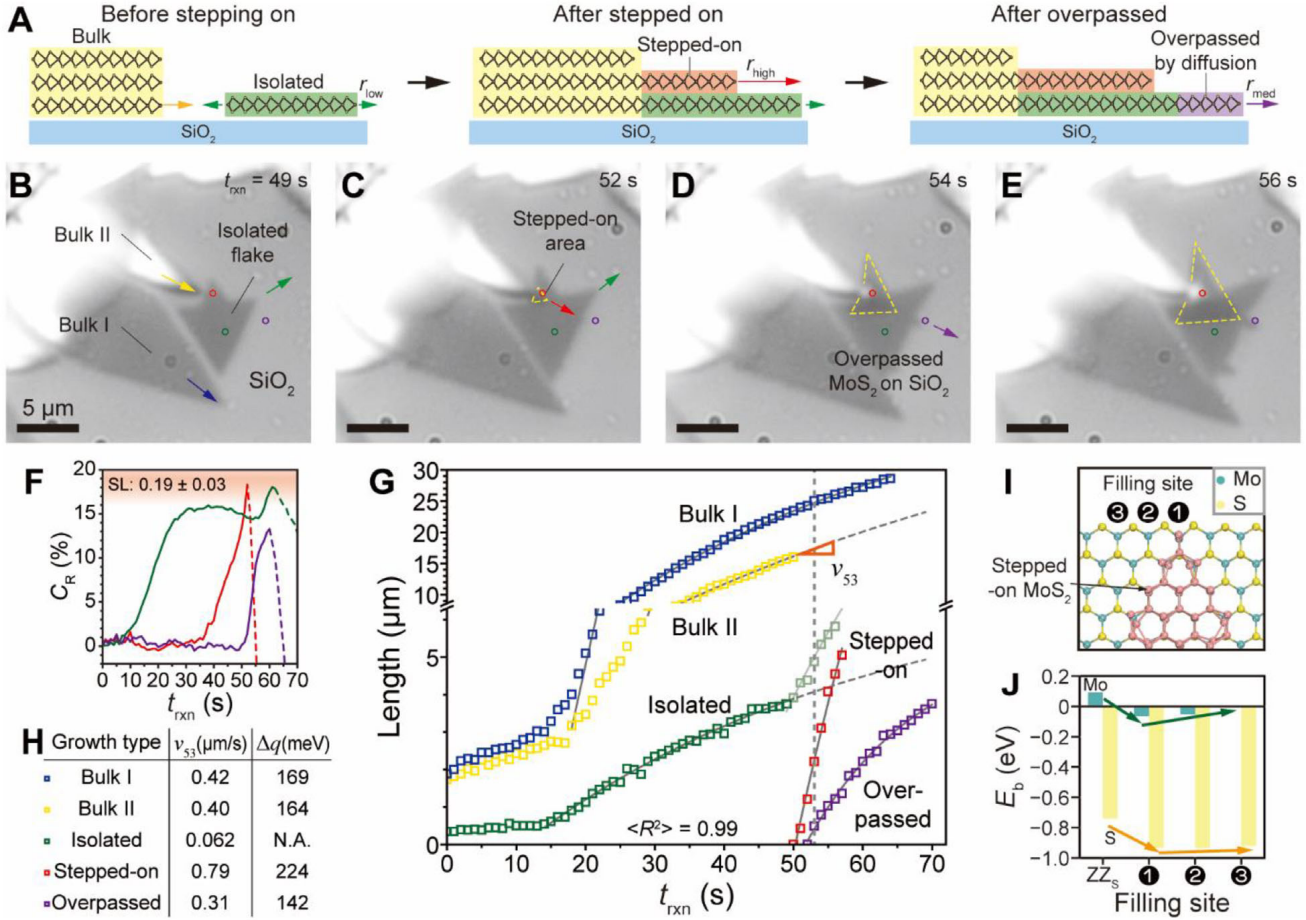
Substrate‐dependent, diffusion‐driven growth. A) Schematic representation of bulk MoS_2_ growth as it steps onto and overpasses an isolated MoS_2_ flake. B–E) Time‐sequenced optical images depicting different growth stages: (B) approaching the isolated flake, (C) stepping‐on the flake, and (D,E) the overpassing stages. Note that tone and contrast of these images have been adjusted, and purple circle is intentionally slightly offset to enhance visual clarity. Scale bars: 5 µm in all images. F) *C*_R_ (%) values corresponding to the regions indicated in (B–E), with the shaded area representing the SL *C*_R_ range. Dashed lines indicate regions where *C*_R_ exceeds the few‐layer regime. G) Time‐dependent growth kinetics of bulk I, bulk II, and the isolated, stepping‐on, and overpassing MoS_2_ regions, with dashed lines indicating projected growth trends. H) Table summarizing *ν*_53_ and Δ*q* values for each growth type. I) Atomic‐scale depiction of the filling site positions for the stepped‐on MoS_2_ overlayer (pink). J) Distance‐dependent binding energy *E*_b_ for Mo and S in isolated MoS_2_ and the stepped‐on MoS_2_ overlayer.

The difference in diffusion rates becomes apparent when examining the growth rate (*v*) of bulk MoS_2_ edges as they overpass an isolated MoS_2_ grain during growth. **Figure** **2**A presents a schematic representation of bulk MoS_2_ (yellow) expanding over an isolated MoS_2_ grain (olive) on two different substrates—MoS_2_ (orange) and SiO_2_ (purple). The growth process is categorized into three distinct stages: the initial stage, the stepping‐on stage, and the overpassing stage. Notably, in the final panel, the overpassed MoS_2_ remains unconnected to the bulk II, indicating no direct physical attachment between them.

Figure 2B–E show time‐dependent optical images capturing the growth process, while Video S2 (Supporting Information) provides a full sequence of the event. Initially, bulk MoS_2_ structures I and II coexist with the isolated flake (Figure 2B). As growth progresses, bulk II extends over the isolated flake, forming an overlayer (Figure 2C). During this stepping‐on process, a distinct triangular shape emerges, outlined by a yellow dashed line in Figure 2D. Eventually, the overpassed MoS_2_ extends beyond the isolated flake without retaining a direct physical connection.

The variation in layer thickness was tracked using the *C*_R_ (%) value, an in situ method for assessing structural changes^[^ ^21^ ^]^ (see Experimental Section). Figure 2F presents *C*_R_ (%) trends corresponding to the color‐coded positions in Figure 2B–E. Initially, the green‐marked position (representing the isolated flake) exhibits a single‐layer (SL) structure, while the red and purple positions gradually become from SL to ML (dashed lines) at later stages. These findings provide direct evidence that precursor diffusion occurs more efficiently on the MoS_2_ surface, leading to faster growth compared to diffusion on SiO_2_.

Analysis of the growth kinetics from Video S2 (Supporting Information) revealed distinct growth rates for bulk II on SiO_2_ and MoS_2_ surfaces. Figure 2G displays a graph of growth length *G*(*t*) as a function of *t*_rxn_ for the vertices of bulk I, bulk II, the isolated grain, stepping‐on bulk II, and overpassing bulk II. While most curves follow a single‐exponential growth trend, the isolated grain exhibits a double‐exponential growth pattern starting at ≈50 s. This behavior results from the combined growth of the initially isolated grain and the newly overpassed region. The observed growth curves are modelled using a self‐exhausting exponential function, which accounts for the precipitation of MoS_2_ from SODE. *G*(*t*) is expressed as follows:^[^ ^21^, ^41^ ^]^

(2) $$G\left(t\right)=v_{0}\tau \left(1-\exp\left(\frac{-\left(t-\varphi \right)}{\tau }\right)\right)$$

where *v*_0_ is the onset growth rate (µm/s), and *τ*, *t*, and *φ* represent the growth time constant, reaction time, and growth time delay in seconds, respectively. These parameters of each curve are detailed in Table S1 (Supporting Information). The growth curves are simulated with Equation (2), with high simulation accuracy as indicated by the <*R* ^2^> value in Figure 2G. Additionally, the growth rate at a specific time *t* (*v_t_*) can be used to compare the growth kinetics. Based on the projected *v*_53_ value for the isolated grain (*v*_53,isolated_ = 0.062 µm s^−1^), the projected *v*_53_ for bulk II (shown by the gray extended curve) is ≈ 0.40 µm s^−1^. However, for the stepping‐on case, the growth rate increases to ≈ 0.79 µm s^−1^, and after overpassing, the projected *v*_53_ (*v*_53,overpassed_) drops to ≈ 0.31 µm s^−1^. This suggests that the stepping‐on area exhibits the fastest growth rate among the three cases. Furthermore, using the *v*_53_ values, we calculate the difference in activation energy for diffusion, Δ*q* = *q*_overpassed_ − *q*_isolated_ (Figure 2H, see Note S1, Supporting Information for full explanation). Δ*q* is expressed as follows:

(3) $$\Delta q=k_{B}T\ln\left(v_{53,\mathrm{overpassed}}/v_{53,\mathrm{isolated}}\right)$$

where *k*_B_ is the Boltzmann constant (8.617 × 10^−5^ eV·K^−1^), and *T* is the absolute temperature. The experimentally obtained Δ*q* (Figure 2H) from the comparison of the overpassed and isolated cases is 142 meV.

### 2.3 Kinetic and Thermodynamic Insights into MoS_2_ Growth using DFT Calculations

To investigate the driving force behind the overpassing growth mechanism following the stepping‐on of MoS_2_, we performed DFT calculations^[^ ^42^, ^43^, ^44^, ^45^ ^]^ (see Experimental Section). In this analysis, we compared the adsorption strength of Mo and S on the zigzag S (ZZ_S_) edges, both with and without an overlying MoS_2_ flake. As shown in Figure 2I, we examined how the adsorption strength of Mo and S varies with different binding positions relative to the MoS_2_ flake, allowing us to explore the distance‐dependent influence. Our results indicate that, when MoS_2_ is stepped on by another MoS_2_ flake, the adsorption strength of Mo and S increases, with more negative binding energy values compared to the case without the stepped‐on MoS_2_, as illustrated in Figure 2J. Additionally, the adsorption strength weakens gradually as the distance from the MoS_2_ flake increases.

**Figure 3:**
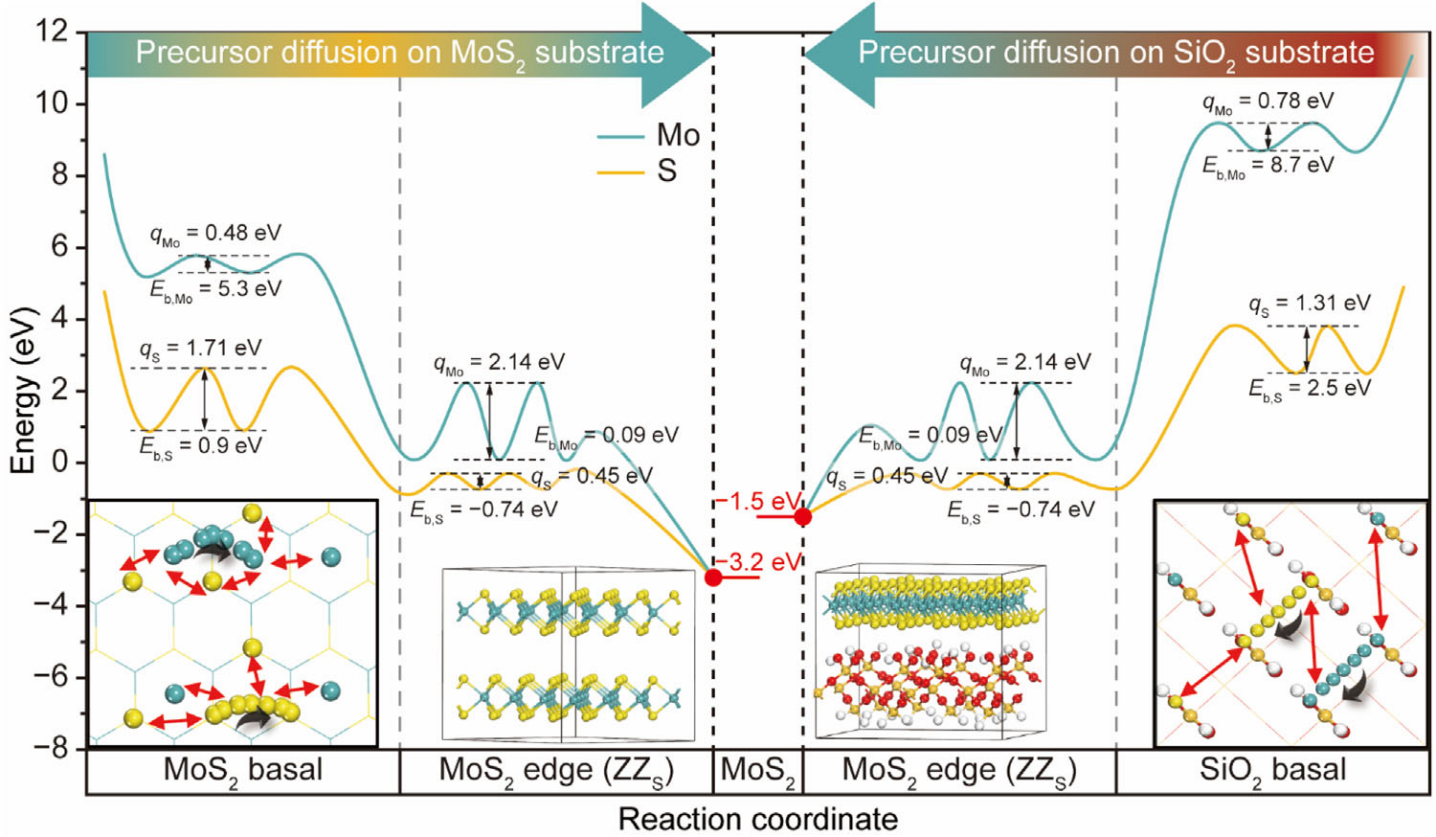
Energy profiles of MoS_2_ growth, including diffusion on MoS_2_ and SiO_2_ substrates. The diagram shows the adsorption energy (*E*_b_) and diffusion energy barriers (*q_i_*) for Mo and S, comparing their diffusion behaviors on MoS_2_ (left) and SiO_2_ (right) surfaces and edges. The outer and inner insets illustrate the diffusion effects of adsorbed Mo and S on each substrate, as well as the interface between the MoS_2_ layer and the SiO_2_ substrate during growth.

To better understand the substrate‐dependent MoS_2_ growth process, both thermodynamic and kinetic factors are systematically investigated.^[^ ^31^ ^]^ **Figure** **3** presents an energy profile comparing MoS_2_ growth on MoS_2_ (left panel) and SiO_2_ (right panel). Both Mo and S atomic sources show positive adsorption energy (*E*_b_) values on MoS_2_ and SiO_2_ surfaces, indicating that Mo and S can diffuse without forming strong interactions with the surfaces. However, the potential energy surfaces for MoS_2_ and SiO_2_ feature non‐uniform potential wells and barriers (see Figure S2A–D for detailed energetics along the reaction coordinates, Supporting Information). The depth of these wells and the height of these barriers (*q_i_*) increase as the *E*_b_ values for Mo and S strengthen on MoS_2_ and SiO_2_ surfaces. While both Mo and S have positive *E*_b_ values, S, having a relatively stronger *E*_b_, also experiences a higher potential barrier. Consequently, S exhibits a higher *q_i_* than Mo on both MoS_2_ and SiO_2_ surfaces.

The *q_i_* values for Mo and S are 0.48 and 1.71 eV on the MoS_2_ surface, and 0.78 eV and 1.31 eV on the SiO_2_ surface, respectively. These *q_i_* values were derived by considering the diffusion of individual Mo or S atoms due to computational limitations. However, as illustrated in the outmost insets, interactions with neighboring atoms can influence the *q_i_* values. On the MoS_2_ surface, the adsorption sites for Mo and S are in close proximity, allowing mobile Mo or S atoms to interact with adjacent atoms, which could reduce their *q_i_* values. Conversely, on the SiO_2_ surface, the adsorption sites for Mo and S are farther apart, minimizing the impact of neighboring atoms on the *q_i_* values. This difference in *q_i_* values serves as a kinetic factor that affects the growth rate of MoS_2_ flakes on MoS_2_ and SiO_2_ substrates.

The experimental Δ*q* between the isolated and the overpassed MoS_2_ is 142 meV, as specified in Figure 2H. For the DFT‐calculated value, we averaged the contributions of Mo and S based on the nominal 1:2 stoichiometry. Therefore, *q*_Mo,MoS2_ and *q*_S,MoS2_ are 0.48 and 1.71 eV, respectively, yielding an average value <*q_i_*_,MoS2_> = 1.30 eV. For SiO_2_, *q*_Mo,SiO2_ and *q*_S,SiO2_ are given by 0.78 and 1.31 eV, respectively, with an average value <*q_i_*_,SiO2_> = 1.13 eV. The theoretically predicted Δ*q* is ≈0.167 eV (i.e., 1.3–1.13 eV), which agrees well with the aforementioned experimental value.

Conversely, MoS_2_ edges exhibit lower *E*_b,_ *_i_* and *q_i_* compared to the basal plane. Specifically, we examined ZZ_S_ edges which are formed at excess S CVD condition. At the ZZ_S_ edge, S has a negative *E*_b_ of −0.74 eV, while Mo has a weaker *E*_b_ of 0.09 eV but a notably high *q_i_* of 2.14 eV. The strong *E*_b_ of S and the high *q_i_* of Mo suggest that once these atoms reach the MoS_2_ edges, they become stably incorporated into the growing MoS_2_ flake. As the flake continues to expand, it forms an interface with the underlying MoS_2_ or SiO_2_ substrate. The thermodynamic stability of this interface plays a crucial role in determining the MoS_2_ flake growth rate. The interaction energy between the MoS_2_ flake and the substrate is −3.2 eV on MoS_2_ and −1.5 eV on SiO_2_, indicating that MoS_2_ flakes adhere more strongly and exhibit greater stability on MoS_2_ surfaces. Considering both kinetic and thermodynamic factors, MoS_2_ flake growth proceeds more efficiently on MoS_2_ substrates than on SiO_2_. Additionally, sodium is known to facilitate the incorporation of Mo and S by lowering *q_i_*.^[^ ^30^ ^]^ Moreover, the observed binding energy trends with the substrate suggest a tendency toward VLS growth modes near the edges.^[^ ^21^, ^24^ ^]^

### 2.4 Diffusional SODE‐Promoted MoS_2_ Growth Behaviors

**Figure 4:**
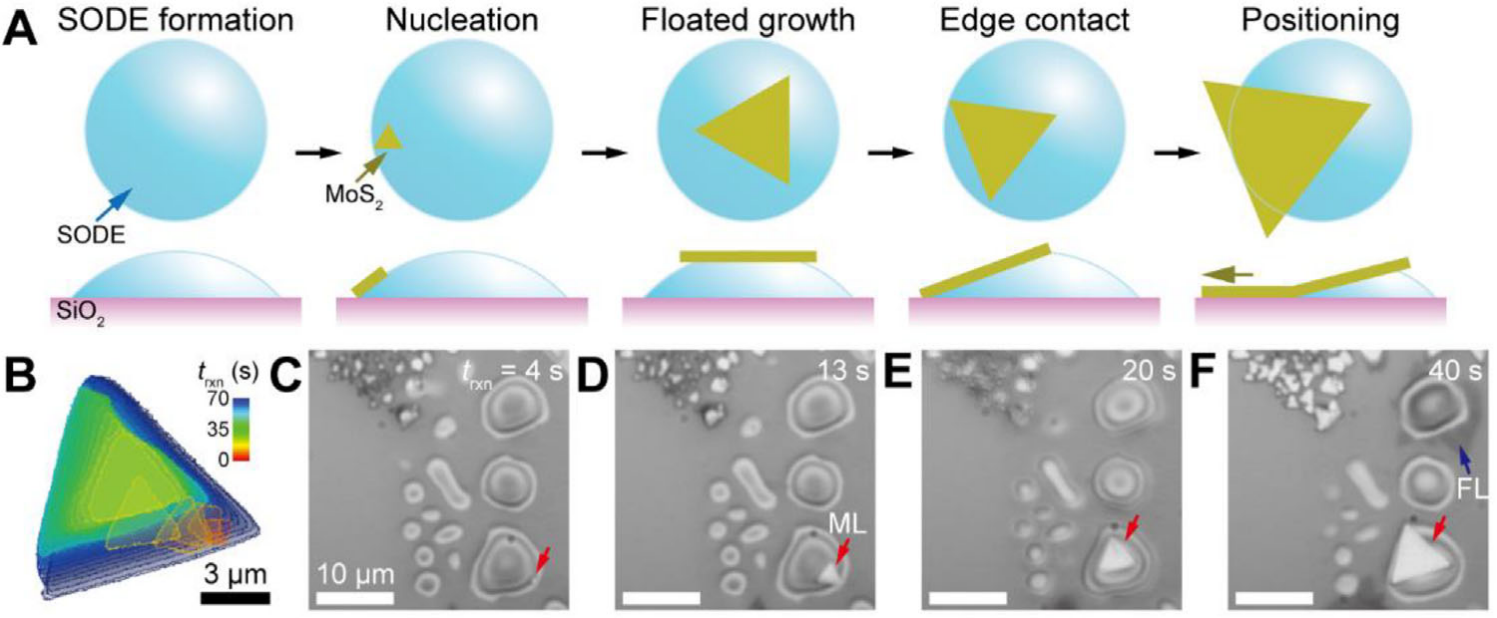
Growth behavior of a floating grain on a stationary large SODE on the SiO_2_ surface. A) Schematic illustrating the growth mode of triangular ML MoS_2_ as *t*_rxn_ progresses. B) Growth contour map showing the floating triangular ML MoS_2_ captured at 2‐s intervals. C–F) Time‐lapse optical images for the formation of the floating ML MoS_2_ as well as FL MoS_2_ on top of the stationary SODE.

The weakened interaction between MoS_2_ and the SiO_2_ substrate, mediated by a relatively larger amount of SODE compared to the previous case, facilitates the formation of ML flakes that exhibit significant rotational drifting. **Figure** **4**A illustrates a schematic of ML MoS_2_ growing on a large SODE. As SODE forms, MoS_2_ nucleation initiates at the three‐phase interface (SODE/argon/substrate), as shown in the second panel. This is likely driven by localized higher surface energy at this site, consistent with previous reports.^[^ ^46^ ^]^ The growing MoS_2_ grain then floats atop the SODE, and upon reaching a sufficient size, its edges anchor to the substrate, transitioning to anchored growth.

Figure 4B presents a growth contour map of the floating MoS_2_ grain on SODE, clearly illustrating its profound floating and rotational growth behavior (see Video S3, Supporting Information for the complete movie clip), compared to the case of Figure 1B. Figure 4C–F display *t*_rxn_‐dependent optical images of the ML flake (red arrow) growing on a stationary SODE, which is a few tens of nanometers thick, as evidenced by its concentric interference patterns.^[^ ^47^ ^]^ A similar growth behavior is observed for the few‐layer (FL) MoS_2_ (blue arrow) growing along the edges of the SODE (Figure 4F). These findings reinforce the idea that MoS_2_ decoupling from the SiO_2_ substrate promotes random rotation and floating during growth. This behavior contrasts with NaCl‐assisted MoS_2_ growth,^[^ ^24^ ^]^ in which NaCl remains as the top layer.

The growth of MoS_2_ from large molten SODE closely resembles 2D Czochralski growth^[^ ^31^ ^]^, a process traditionally used for producing single crystals of materials such as silicon, germanium, metals, salts, and synthetic gemstones from the molten phase. Microscopically, the origin of the Czochralski MoS_2_ growth is likely to associate with the scooting mechanism observed with small SODEs—which drives MoS_2_ growth by simultaneous lateral movement and precipitation along the crystal edge.^[^ ^21^, ^48^ ^]^

**Figure 5:**
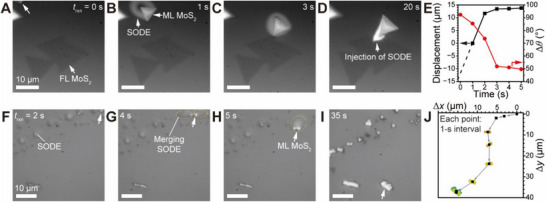
Drifting ML grains on floating SODE, resembling a “flying nimbus” on the SiO_2_ surface. A–D) Time‐lapse optical images capturing the long‐range motion of ML MoS_2_ on mobile SODE. E) Graph depicting the translational (left) and rotational (right) movements of the grain on SODE as a function of *t*_rxn_. F–I) Sequential optical images illustrating the extended travel of growing ML MoS_2_ on equally growing and traveling SODE on the substrate. J) Spatial trajectory plot of the moving MoS_2_, overlaid with the evolving shape of the growing MoS_2_.

Additionally, ML MoS_2_ exhibits long‐distance drifting on floating SODE, resembling a “flying nimbus”. Videos S4 and S5 (Supporting Information) capture two intriguing instances of floating ML MoS_2_ interacting with SODE. **Figure** **5**A–D present time‐sequenced optical images showcasing the long‐range movement of a triangular bulk MoS_2_ flake on SODE. Initially positioned at the corner (Figure 5A), the MoS_2_ grain drifts rapidly at a speed of 11.7 µm s^−1^, traveling ≈ 14 µm before settling near the target FL MoS_2_ aggregates (Figure 5C). Upon arrival, it continues growing into bulk MoS_2_ at the expense of SODE (Figure 5D). During this motion, the SODE surrounding the drifting MoS_2_ flake emits a luminous halo, attributed to light reflection under dark illumination due to partial screening of the objective lens near the viewport rim of ICVDM.^[^ ^21^ ^]^ Figure 5E illustrates both the large displacement and large rotational movement observed during this drift.

The mobility of liquid SODE facilitates the agglomeration of nearby SODE and supports the formation of ML MoS_2_. Figure 5F–I present time‐sequenced optical images capturing the growth of a MoS_2_ flake on mobile SODE as it merges with adjacent SODE during the reaction. Initially, small ML MoS_2_ clusters (Figure 5F) drift and coalesce with nearby SODE, continuing to move and grow into ML MoS_2_ aggregates (Figure 5F–H) until all available SODE is depleted (Figure 5I). Given the high carrier gas flow rate (180 standard cubic centimeters per minute (sccm)), these findings indicate that SODE exhibits weak adhesion to the SiO_2_ substrate, enabling MoS_2_ to drift over extended distances.

### 2.5 Effect of Mobile SODE on Continuous Growth

Up to this point, we have explored the diffusion dynamics and substrate influence of precursor/SODE during MoS_2_ growth. Now, we turn our attention to the role of SODE in sustaining continuous MoS_2_ growth. The presence of sodium in reaction significantly impacts the growth process. Figure S3A–F (Supporting Information) illustrate the sulfurization of drop‐cast MoO_3_ precursor under similar conditions but without SC. In the absence of sodium, MoS_2_ growth remains confined to the drop‐cast MoO_3_ region (see Note S2, Supporting Information for a detailed explanation of Figure S3, Supporting Information).

**Figure 6:**
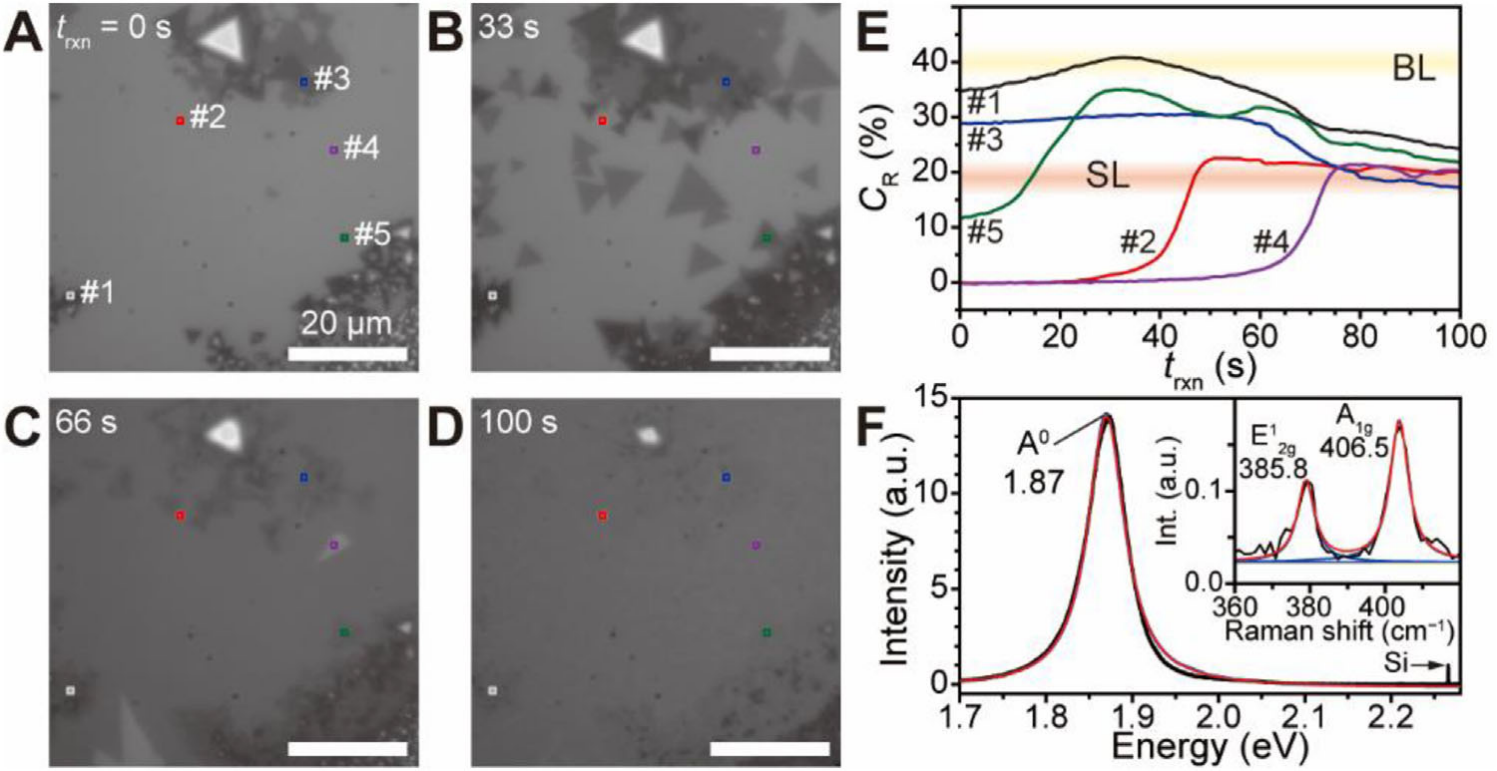
Formation of continuous SL MoS_2_ facilitated by SODE diffusion. A–D) Time‐lapse optical images showing the transition from individual grains with different number of layers to continuous SL MoS_2_. E) *C*_R_ (%) trend obtained from time‐lapse optical images at five different positions from (A–D). Yellow and red bands indicate SL and BL regimes. F) PL spectrum of the resulting continuous MoS_2_ film. Inset: Corresponding Raman spectrum with interpeak separation of 20.7 cm^−1^. The red curves present the deconvoluted Lorentzian shapes.

In contrast to the absence of SC, the presence of SC enhances the uniform growth of MoS_2_ across the substrate. Growth with SC‐containing metal precursor promotes delocalized MoS_2_ growth, likely driven by the diffusion of precursor‐laden SODE. Video S6 (Supporting Information) provides a movie clip of this process. **Figure** **6**A–D show *t*_rxn_‐dependent optical images demonstrating the formation of continuous SL MoS_2_ from individual grains with varying layer numbers. Initially, Figure 6A shows MoS_2_ grains with different layer counts spread across the substrate. As the reaction progresses, SL grains (Figure 6B) begin to grow not only at the edges of the existing grains but also across ‘empty’ spaces on the SiO_2_ substrate. These grains (Figure 6C) eventually merge into a continuous sheet, and after some time, the layer count converges to SL MoS_2_ (Figure 6D). The formation of a layer‐even continuous MoS_2_ structure is further supported by *C*_R_ trends across the area (Figure 6E). Initially, *C*_R_ values vary at different spots (as shown in Figure 6A–D), but by the end of the reaction, these values converge to SL MoS_2_, indicating the rapid diffusion of SODE across the bare substrate to facilitate MoS_2_ growth. This leads to the uniform growth of SL MoS_2_. The convergence of the layer number is likely attributed to the inherent instabilities of the edges compared to the basal plane. Overall, these findings highlight the critical role of molten sodium in accelerating the growth process due to its high mobility.

Figure 6F and its inset show the photoluminescence (PL) and Raman spectra of the resulting MoS_2_, respectively. The PL spectrum indicates that the intensity of the continuous MoS_2_ is approximately 14 times higher than that of the Si band at 520.9 cm^−1^,^[^ ^49^ ^]^ indicating high‐quality MoS_2_.^[^ ^50^ ^]^ The deconvolution of the PL spectrum best fits with a single Lorentzian curve at 1.87 eV, corresponding to the neutral exciton (A^0^) band of MoS_2_.^[^ ^51^, ^52^, ^53^ ^]^ Raman measurements reveal two characteristic peaks of MoS_2_ at 385.8 and 406.5 cm^−1^, attributed to the in‐plane E^1^_2g_ mode and out‐of‐plane A_1g_ mode vibrations, respectively.^[^ ^54^ ^]^ The interpeak separation, which serves as an indicator of the layer number, is 20.7 cm^−1^, confirming the formation of SL MoS_2_.^[^ ^49^, ^54^, ^55^ ^]^ These results strongly support the hypothesis that the mobility of SODE facilitates the uniform growth of MoS_2_, consistent with previous studies.^[^ ^1^, ^30^ ^]^

However, the massive diffusion of submicrometer‐sized SODEs can prevent SL formation of MoS_2_ by forming ML at the edges. Figure S4A–D (Supporting Information) shows time‐lapse optical images of MoS_2_ grains growing due to the diffusion of SODEs across the substrate (see Video S7, Supporting Information for the full movie clip). In these images, several SODEs begin to diffuse toward the edges of the existing MoS_2_ grains. The red dashed enclosure denotes the boundaries of the diffused SODEs as the reaction progresses. Figure S4E (Supporting Information) shows the broadened boundary of the consumed SODE over time. As a result of the massive diffusion of SODEs, MoS_2_ forms ML near the rim, contrasting with the inner centroid. Additionally, this result emphasizes the importance of maintaining an adequate grain density per SODEs to create a uniform MoS_2_ film.

Overall, SODEs exhibit size‐dependent diffusive behaviors which promote the different morphologies of grain. When its size is smaller than the optical detection limit of ICVDM system (i.e., 620 nm considering monochromatic light source and lens magnification used in our experiment), smaller SODE having high diffusion rate owing to smaller size predominantly catalyzes SL grain by precipitating the impregnated MoS_2_ laminates, as evident in Figure 6 and the cases in our previous work.^[^ ^21^ ^]^ It is noteworthy that we did not observe any motions for SL‐ and FL MoS_2_. This might be related to the conforming nature of SL and FL MoS_2_ to the surface morphologies,^[^ ^56^ ^]^ compared to ML MoS_2_.

When the size of SODE belongs to the regime for the optical detection, this larger SODE promotes FL or ML grains owing to the faster rate of MoS_2_ precipitation and slower diffusion rate (i.e., the cases of Figures 1, 4, and 5). Furthermore, when the size of coalesced SODE is comparable or greater than that of grain, and SODE promotes ML MoS_2_ growth on its surface rather than the interface between SODE/substrate, the growth of the grain displays floating behaviors. These are the opposite cases for the nucleus grown from the interface between SODE/substrate (FL MoS_2_ growth in Figure 4), FL MoS_2_ does not move at all. In addition, we witnessed that SODE morphologies are different (i.e., droplet vs. wetting for Figures 1, 4, and 5, respectively), although similar conditions are utilized. These seem to originate from the minute interaction difference with the substrate. Recently, our group reported that pH of precursor solution as well as substrate hydrophilicity plays a crucial role for the formation of controlled growth of continuous MoS_2_.^[^ ^38^ ^]^ Therefore, we speculate that the morphology variations of SODE is dependent on a localized hydrophilicity of the substrate.

## 3 Conclusion

In this study, we investigated the diffusion‐ and substrate‐mediated growth kinetics of MoS_2_, promoted by precursor and SODE, both experimentally and theoretically. Real‐time observations of MoS_2_ growth via ICVDM reveal the diffusion pathway of the precursor and SODE. SODE follows a conventional surface growth process, diffusing from adatoms to grains and edges. Furthermore, SODE shows a preference for the interface between MoS_2_ and SiO_2_, and promotes the weak interaction with MoS_2_, leading to translational and rotational movement behaviors. This MoS_2_ floating on a large amount of SODE exhibits high mobility across the SiO_2_ surface, showing long‐range drifting behavior. A comparison of the stepping‐on and overpassing MoS_2_ growth behaviors on an isolated MoS_2_ reveals substrate growth kinetics mediated by diffusion. This prompted a detailed investigation using DFT calculations to examine the binding and activation energies of diffusion for each precursor on MoS_2_ and SiO_2_ surfaces and edges, revealing MoS_2_ as the preferred substrate for both thermodynamic and kinetic reasons. Controlling diffusional behaviors of SODE can help minimize mismatched epitaxial growth, and controlling the mobility and size of molten metals would be advantageous for large‐area, high‐quality growth of various TMCs.

## 4 Experimental Section

### Materials and Instrumentation

Molybdenum (VI) oxide (purity ≥ 99.5%) and sulfur (purity ≥ 99.5%) were obtained from Merck, while SC (purity ≥ 98%) was sourced from TCI. Deionized (DI) water with a resistivity exceeding 18 MΩ was used to prepare the MoO_3_/SC solution. High‐purity argon (≥99.99%) was supplied by Donga Gas (Republic of Korea) and served as the carrier gas. The acquired Si wafer (285 nm thick SiO_2_/Si substrate, ShinEtsu) was cut into 1 × 1 cm^2^ pieces, cleaned sequentially with methanol, acetone, and isopropanol, and dried under a nitrogen stream. AFM height images were obtained in tapping mode using an NX10 AFM (Park Systems), following literature procedures.^[^ ^37^ ^]^ Al‐coated silicon cantilevers (force constant: 37 N m^−1^, resonance frequency: 300 kHz, ACTA‐20, App Nano) were used. Typically, 512 × 512 pixel images were captured at a scanning speed of 0.2 Hz. The XEI software (Park Systems) was employed to flatten topographies using a polynomial along the fast scan axis. Raman and PL measurements in a back‐scattering configuration were performed with either a custom‐built Raman system^[^ ^57^, ^58^ ^]^ or the XperRam C (Nanobase), using 532‐nm excitation unless otherwise specified. The spectrum was calibrated to the 520.9 cm^−1^ Si band.^[^ ^49^, ^57^, ^58^ ^]^

### MoS_2_ Growth Observed in *Operando*

All CVD growth processes were carried out using an ICVDM.^[^ ^21^ ^]^ However, compared to the ICVDM prototype presented in the previous work,^[^ ^21^ ^]^ the current setup—referred to as ICVDM Mk. I—features numerous upgrades that enhance measurement capabilities. For general accessibility, Note S3 (Supporting Information) has been added to provide detailed information about the ICVDM system, including procedural guidelines and descriptions of several custom‐made components that are essential for real‐time measurements. In brief, unlike the earlier prototype, the Mk. I setup primarily employs a long working‐distance 50× objective lens (LMPlanFL, working distance: 10.6 mm, N.A.: 0.5; Olympus), with the optional use of a 100× objective (SLMPlan N, working distance: 7.6 mm, N.A.: 0.6). These lenses offer optical resolutions of approximately 620 nm (for 50×) and 520 nm (for 100×), respectively, under deail diffraction‐limited conditions. This resolution *d* is determined by the Abbe's formula:^[^ ^59^ ^]^

(4) $$d=\lambda /\left(2\times N.A.\right)$$

where *λ* is the wavelength. 620‐nm bandpass filter is used on the excitation side for the determination of *C*_R_. Additionally, although the thinnest available coverslip (25 mm diameter, 80 µm thickness) is used as the transparent viewport for the mini‐CVD system, it introduces slight optical aberrations that slightly degrade the resolution. The growth contour map and subsequent kinetic analysis, based on a movie clip, were conducted using freeware ImageJ as described in a previous report.^[^ ^21^ ^]^

### Sample Preparation

The MoO_3_/SC dispersion was prepared by dissolving 20 mm MoO_3_ in 1 wt.% SC within 35 mL of distilled water. This mixture underwent bath sonication for 1 h, followed by centrifugation at 5000 *g* (*g* = 9.8 m s^−2^) for 30 min, after which approximately 80% of the supernatant was collected. The MoO_3_/SC dispersion was applied to the substrate and spin‐coated at 3,000 rpm for 80 s. Subsequently, the MoO_3_/SC‐coated substrate was placed in a mini‐CVD system. A separate 100 µL portion of MoO_3_/SC solution was spin‐coated at 5,000 rpm for 1 min onto an O_2_ plasma‐treated substrate unless otherwise noted, which was then positioned inside a mini‐CVD crucible. Additionally, 1,000 mg of sulfur, contained in an alumina crucible, was placed inside the sulfur tube furnace. The parameters for the mini‐CVD system, charge‐coupled device (CCD), chalcogen tube furnace, and flow controller were set accordingly. The mini‐CVD and sulfur temperatures were ramped to 830 and 290 °C, respectively, under a 180‐sccm argon flow unless specified otherwise. The growth process was maintained at the target temperature for 20 min, with ramping rates of 20 °C min^−1^ for sulfur and 100 °C min^−1^ for the MoO_3_ precursor. After the growth phase, the system was allowed to cool naturally to room temperature.

### C_R_ Measurement

This measurement is employed to determine the number of MoS_2_ layers in real‐time using a CCD, as described in the literature.^[^ ^21^, ^57^, ^60^ ^]^ A 620‐nm bandpass filter was used on the excitation side, and an LED power of 6 µW cm^−2^ provided sufficient optical image contrast. Objective lenses with 100× and 50× long working distances were utilized. *C*_R_ was obtained using the following equation:

(5) $$C_{R}=\left(R_{S}-R_{0}\right)R_{0}$$

where *R*_S_ and *R*_0_ denote the reflection intensities of MoS_2_ sample on a substrate and bare substrate, respectively. For real‐time determination, reflection intensities of bare and sample regions obtained from the CCD interface are utilized.

### DFT Calculation

All ab initio calculations were carried out using the Vienna ab initio Simulation Package (VASP 5.4.4).^[^ ^42^, ^43^, ^44^, ^45^ ^]^ The projector augmented wave (PAW) method^[^ ^44^, ^45^, ^61^, ^62^ ^]^ was utilized, and exchange‐correlation interactions were treated with the Perdew–Burke–Ernzerhof (PBE) functional^[^ ^63^ ^]^ under the generalized gradient approximation (GGA). Integration within the Brillouin zone was performed using the Monkhorst–Pack scheme, with 6 × 6 × 1 and 3 × 3 × 3 *k*‐point meshes for the geometry optimization of bulk MoS_2_ and SiO_2_ structures, respectively. For surface calculations, the MoS_2_ basal plane was sampled using a 2 × 2 × 1 *k*‐point mesh, while the MoS_2_ edge structure was sampled with a 3 × 1 × 1 *k*‐point mesh. The SiO_2_ substrate was modeled based on the most stable (001) surface, fully OH‐terminated,^[^ ^64^ ^]^ with its Brillouin zone sampled using a 2 × 2 × 1 *k*‐point mesh.^[^ ^65^ ^]^ The dispersion correction method (DFT‐D3) was employed to account for non‐bonding interactions in the entire system.^[^ ^63^, ^66^ ^]^ Lattice constants and internal atomic positions were fully optimized using a plane‐wave cutoff energy of 500 eV and spin‐polarized calculations, with convergence achieved when residual forces were below 0.04 eV Å^−1^. Additionally, the activation energies for the diffusion of Mo and S atoms on MoS_2_ and OH‐terminated SiO_2_ (001) surfaces were studied using the climbing image nudged elastic band (Cl‐NEB) method.^[^ ^67^ ^]^

## Conflict of Interest

The authors declare no conflict of interest.

## Author Contributions

J.O., Y.K., and J.H.S. contributed equally to this work. J.O. performed experiments and analyzed data. Y.K. performed experiments and analyzed data. J.H.S. and Y.H.K. performed theoretical calculations. J.S. performed experiments. S.U.L. supervised theoretical calculations. S.‐Y.J., S.U.L., and Y.K. co‐wrote the manuscript. All the images/artwork/photos that appear in the manuscript and Supporting Information file were created by the authors of this manuscript.

## Supporting information



Supporting Information

Supplemental Video 1

Supplemental Video 2

Supplemental Video 3

Supplemental Video 4

Supplemental Video 5

Supplemental Video 6

Supplemental Video 7

## Data Availability

The data that support the findings of this study are available in the supplementary material of this article.
