# Supplementary material for: Diffusion and Surface Effects on Sodium‐Promoted MoS2 Growth Observed in Operando
Source: Small Methods. 2025 Aug 7;9(9):e00813. doi: 10.1002/smtd.202500813 (PMC12464655; doi:10.1002/smtd.202500813)
Supplement: Supplementary file 1 — Supporting Information [file SMTD-9-e00813-s008.docx]

Supporting Information

Diffusion and Surface Effects on Sodium-Promoted MoS_2_ Growth Observed in *Operando*

Jehyun Oh,^1,†^ Yoonbeen Kang,^1,†^ Jae Hun Seol,^2,†^ Yong Hui Kim,^2^ Jinyoung Seo,^1^ Sang Uck Lee,^2,^* and Sang-Yong Ju^1,^*

J. Oh, Y. Kang, J. Seo, S.-Y. Ju

Department of Chemistry, Yonsei University, Seodaemun-Gu, Seoul 03722, Republic of Korea

E-mail: syju@yonsei.ac.kr

J. H. Seol, Y. H. Kim, S. U. Lee

School of Chemical Engineering, Sungkyunkwan University, Suwon 16419, Republic of Korea

E-mail: suleechem@skku.edu

^†^J. Oh, Y. Kang, and J. H. Seol contributed equally to this work.

**Note S1: Calculation of** **Δ*q***

To calculate Δ*q* in Figure 2H, we take into account how the activation energy for diffusion of each species *q_i_* contributes to the activation energy of each atomic site *E*_fill,_*_i_* which in turn governs the filling rate *r*_fill,_*_i_*. The *r*_fill,_*_i_* value is defined as follows:^[1-3]^

$r_{fill, i, MoS_{2}}=(\frac{P_{i,MoS_{2}}}{\sqrt{2\pi M_{i}RT}})N_{A}A_{\mathrm{site}}exp(-\frac{E_{fill,i}}{k_{B}T})$ (S1)

$r_{fill, i, SiO_{2}}=(\frac{P_{i,SiO_{2}}}{\sqrt{2\pi M_{i}RT}})N_{A}A_{\mathrm{site}}exp(-\frac{E_{fill,i}}{k_{B}T})$ (S2)

where *P_i_* and *M_i_* represent the pressure and molar mass of species *i*, respectively, *R* is the gas constant (8.314 J·K^−1^·mol^−1^), *k*_B_ is the Boltzmann constant (8.617 × 10^−5^ eV·K^−1^), and *N*_A_ is the Avogadro constant (6.022 × 10^23^ mol^−1^). The area of collision site *A*_site_ is given by *A*_site_ = 8.65 Å^2^ based on a MoS_2_ lattice.^[4]^ Since *E*_fill,_*_i_* values are the same for a given collision atomic site, the difference in growth rates between *r*_fill,_*_i_*_,MoS2_ and *r*_fill,_*_i_*_,SiO2_ is caused by the differences in *P_i_*_,MoS2_ and *P_i_*_,SiO2_.

On the other hand, the diffusivities of each precursor *D_i_* (where *i* refers to Mo or S) decreases when molecules collide each other on a surface, and is inversely proportional to *P_i_*, as given by the following equation:^[5,6]^

$D_{i}=\sqrt{\frac{{k_{B}}^{3}}{\pi^{3}M_{i}}}\frac{T^{1.5}}{P_{i}a^{2}}$ (S3)

where *a* denotes the lattice size. By dividing eq (S1) and (S2), we obtain the following eq (S4):

$\frac{r_{fill, i, MoS2}}{r_{fill, i, SiO2}}=\frac{P_{i,MoS2}}{P_{i,SiO2}}$ (S4)

*D_i_* are also governed by the following equation:^[7-9]^

*D_i_* = *D*_0,_*_i_* exp(−*q_i_*/*k*_B_*T*) (S5)

where *D*_0,_*_i_* is the initial diffusivity. Based on the growth process shown in Figure 1A, this suggests that the activation energy for diffusion of each species on the MoS_2_ surface is higher than that on the SiO_2_ surface.

Combining eq (S3), (S4), and (S5), we derive eq (S6) as follows:

$\frac{r_{fill, i, MoS_{2}}}{r_{fill, i, SiO_{2}}}=\frac{P_{i,MoS_{2}}}{P_{i,SiO_{2}}}=\frac{D_{i,SiO_{2}}}{D_{i,MoS_{2}}}=\frac{D_{0,i,SiO_{2}}}{D_{0,i,MoS_{2}}}\exp(\frac{q_{i,MoS_{2}}-q_{i,SiO_{2}}}{k_{B}T})$ (S6)

At a given *T* (*i.e.*, 1023 K) and *P_i_*, *v*_53_ for the isolated MoS_2_, which represents the smallest growth rates among those in Figure 2H, is proportional to 0.062 μm/s. By assuming that the initial diffusivities are the same, Δ*q* is described by eq (S7): Δ*q* = *q*_overpassed_ − *q*_isolated_ where *q*_isolated_ denotes the *q* value of the isolated MoS_2_. Since *v*_53_ is proportional to $\boldsymbol{r}_{\mathbf{fill,}\boldsymbol{i}}$ and *q* value is in the numerator of exponential term, we determine Δ*q* from the ratio of *v*_53_. Therefore, Δ*q* is also expressed as follows:

$\Delta q=k_{B}T\ln\left( \frac{v_{53,overpassed}}{v_{53, isolated}} \right)$ (3)

Especially, the experimental Δ*q* between the isolated and the overpassed MoS_2_ is 142 meV, as specified in Figure 2H. For the DFT-calculated value, we averaged the contributions of Mo and S based on the nominal 1:2 stoichiometry. Therefore, *q*_Mo,MoS2_ and *q*_S,MoS2_ are 0.48 and 1.71 eV, respectively, yielding an average value <*q_i_*_,MoS2_> = 1.30 eV. For SiO_2_, *q*_Mo,SiO2_ and *q*_S,SiO2_ are given by 0.78 and 1.31 eV, respectively, with an average value <*q_i_*_,SiO2_> = 1.13 eV. The theoretically predicted Δ*q* is 1,300 meV − 1,133 meV = ~167 meV, which agrees well with the experimental value of 142 meV.

**Note S2: Full explanation of Figure S3**

We examine the growth of MoS_2_ without sodium cholate in the metal precursor droplet. Figure S3A show how the parameters change with *t*_rxn_. In this case, CVD temperature *T*_CVD_ is set to 830 °C while other parameters (*i.e.*, sulfur temperature *T*_S_ and argon flow rate *F*_Ar_) remain similar to those in previous experiments. Figure S3B–S3E display *t*_rxn_-dependent optical images of MoO_3_ droplets as they undergo sulfurization, with the droplet morphologies evolving and beginning to grow MoS_2_ according to the literature.^[1]^ MoS_2_ clearly grows around the edges of the droplet. Figure S3F displays the *C*_R_ trends observed in Figure S3D–S3E, indicating that the number of MoS_2_ layers varies with the distance from the droplet edges. This result indicates that MoS_2_ growth without SC is localized.**Note S3: Detailed Procedural Guidance to operate ICVDM**

Although the initial ICVDM setup—referred to as the ICVDM prototype—was presented in our previous work,^[1]^ we now aim to offer a comprehensive procedural guide for the upgraded configuration, designated as ICVDM Mk. I. To fully benefit from this guide, readers need to first familiarize themselves with the earlier setup (described in the Methods section and Figure S1 of ref. ^[1]^).

This guide is organized into three main parts: (1) the ICVDM Mk. I setup, (2) its operational procedures, and (3) main improvement.

1. ICVDM Mk. I setup

To improve upon the measurement capabilities of the ICVDM prototype,^[1]^ we employed a vibration-isolated optical table and shelving system commonly used in spectroscopic setups (see Figure S5A). This setup minimizes vibrations from sources such as the personal computer (PC) and various controllers. Mounted on this structure are the CVD^[10]^ and microscope systems.

i) Vibration-isolated optical table and shelf

The entire setup is built on a vibration-isolating optical table (dimensions (w×d×h) = 1800 mm × 1200 mm × 800 mm, Daeil systems) coupled with a low-noise air compressor (95 L/min, 550 W, Keyang). Without such isolation, environmental vibrations can be transmitted to the mini-CVD chamber, causing image tremors that are difficult to correct using software like Fiji. Therefore, it is strongly recommended to install all components on a vibration-isolated optical platform.

ii) Flow meter controller, readout, gas lines, and connections

Argon carrier gas flow is managed using a digital mass flow controller (i-300C, Factors) and a readout unit (PMC-4000, Factors) which is PC-compatible via USB. The gas lines primarily consist of 1/4-inch stainless steel tubing, and connections between devices use KF flanges in sizes 25 or 40.

iii) Chalcogen furnace assembly

The chalcogen tube furnace (Lindberg Blue M, 1-inch tube, Thermo Fisher; see Figure S5B) is mounted on two lab jacks (245 mm × 165 mm, Jaeil optical system) and a ball bearing-based linear rail (KGT) to allow precise adjustment of height and position relative to the KF flange inlet of the mini-CVD. A larger furnace was selected to ensure efficient heating of the carrier gas, which helps prevent condensation of chalcogen vapor en route to the mini-CVD. The quartz tube inside the furnace is supported by a pair of custom-made aluminum brackets (see inset of Figure S6), offering slight horizontal flexibility—crucial for aligning the sample’s region of interest (ROI) in the mini-CVD. The furnace’s temperature distribution, based on center-point profiles,^[11]^ is used to determine the temperature at the sulfur boat’s position. The chalcogen sample is positioned just downstream of the furnace at the KF flange joint (Figure S5A). Temperature is regulated via a 3216 Eurotherm controller connected to a PC through a USB interface (Multi-USB, Systembase). The temperature profile—comprising setpoints, ramping rates, and dwell times—is programmed using iTool software.

iv) Mini-CVD chamber on fluorescence microscope

An upright fluorescence microscope (BX43, Olympus) is used for focusing on the mini-CVD sample (TS1000V-17/3, Linkam Scientific) and for potential future spectroscopic integrations. We custom-machined an adapter to mount the mini-CVD onto the microscope‘s XY stage (see Figures S5C and S7), based on drawings for the U-SVRM stage (Olympus) and the mini-CVD chamber.^[12]^ This adapter replaces the original specimen holder and allows for movement of the mini-CVD via the microscope’s coarse/fine adjustment knobs. A 620-nm bandpass filter (10 nm FWHM, Thorlabs) was installed on the excitation path to enable quantitative *C*_R_ measurements.

Several key modifications were also made. The mini-CVD stage lid, originally comprising a lid, fused silica window, and lid insert,^[12]^ was updated with a custom low-profile lid insert (see inset of Figures S5C and S8), which provides a larger field of view and enables easier lens turret rotation during measurements. Furthermore, the original round fused silica window (55 mm × 1 mm) was replaced by an assembly consisting of a donut-shaped quartz disk (outer diameter: 54.5 mm; inner diameter: 12 mm) and a No. 0 cover glass (22 × 22 mm, thickness: 0.08-0.13 mm; Ted Pella, Product No: 260300), bonded using high vacuum grease (Dow Corning) (see inset of Figures S5C and S9).

v) Exhaust and oil bubblers

The exhaust line is connected to a silicon oil-filled bubbler, which is cooled using a cold trap (Eyler), and is followed by a second oil bubbler to provide visual confirmation of gas flow.

2. Detailed procedural guidance to operate ICVDM Mk. I

i) Sample mounting

Initially, a fused silica tube is placed and supported by a pair of custom-made aluminum brackets within the chalcogen furnace assembly. The sulfur crucible—loaded with approximately 1 g of sulfur powder—is then positioned at an appropriate location on the silica tube (typically 16 cm from the center) to reach the desired temperature. Simultaneously, the MoO_3_/SC-coated substrate is mounted inside a mini-CVD crucible. For lenses with relatively short focal lengths, the sample is elevated on a 2-mm-thick sapphire hoist inside the mini-CVD chamber to optimize focus.

ii) Configuring the ICVDM Mk. I hardware

The mini-CVD chamber is sealed in the following sequence: the chamber lid, a 1.0-mm-thick donut-shaped quartz disk, a 0.08-mm-thick cover slip, and a custom-made lid insert. After assembly, the substrate is focused and ROI is identified using long-working-distance objective lenses (100×: SLMPlan N, WD: 7.6 mm, N.A.: 0.60; or 50×: LMPlanFL, WD: 10.6 mm, N.A.: 0.50, Olympus) and CCD camera (Cool SNAP MYO; or Retiga R6, Teledyne Photometrics). Next, the connections between components—specifically from (ii) to (iii) and from (iii) to (iv)—are completed using centering rings between the KF flanges, leading to the sealed chalcogen furnace assembly. For *C*_R_ measurements, a 620-nm bandpass filter is inserted into the excitation path of the microscope. Once the setup is finalized, the mass flow controller/readout, chalcogen furnace, and mini-CVD chamber are activated to regulate the argon carrier gas flow (commonly 180 sccm) and precursor temperatures (typically *T*_CVD_ = 750 °C and *T*_S_ = 250 °C for MoS_2_ growth).

iii) Configuring growth parameters using a PC

Figure S5A contains a screen image on the display. Top display shows optional Raman program and botoom one exhibits multiple windows to set growth parameters. For CCD setting, frame per second (fps, 1.0-2.0 fps) and subsequent exposure time (100-200 ms) and time-lapsed delay are configured in CCD program (i.e., PVCam). The following table lists typical setting parameters. Commonly, save format is TIFF and a series of image is saved in a sequence mode.

Next, *T*_S_ is configured. Temperature controller (3216, Eurotherm) of *T*_S_ is controlled by a designated program (i.e., iTools). Commonly, parameters such as set temperature (750 °C), ramping rate (60 °C/min), and dwelling time (60 min) are programmed. Especially, we utilized the following conversion table for the position 16-cm away from the center and the central position whose temperatures are designated as *T*_S_ and *T*_f,center_.

| *T*_S_ (°C) | 150 | 160 | 170 | 180 | 190 | 200 | 210 | 220 |
| --- | --- | --- | --- | --- | --- | --- | --- | --- |
| *T*_f,center_ (°C) | 540 | 566 | 590 | 614 | 635 | 655 | 675 | 695 |
| *T*_S_ (°C) | 230 | 240 | 250 | 260 | 270 | 280 | 290 | 300 |
| *T*_f,center_ (°C) | 712 | 730 | 750 | 762 | 780 | 795 | 810 | 825 |

For the parameter setting of *T*_CVD_, a designated program (i.e., Link) is utilized. Commonly, parameters such as set temperature (i.e., 750 °C), ramping rate (100 °C/min), and dwelling time (60 min) are set. Finally, a screen capture program (i.e., oCAM in our case) was turned on to record a sequence of *t*_rxn_, *T*_CVD_, *T*_S_, and *F*_Ar_. Overall, one set of ICVDM experiment taking ~30 min *t*_rxn_ generates a series of images with few tens of GB, depending on the numbers of pixels in CCD sensor array (i.e.: Cool SNAP MYO: ~18 GB; Retiga R6: ~32 GB).

3. Main improvement in ICVDM Mk. I

The upgrade is mainly focused on improving the convenience of the ICVDM for user. The change of objective lens from 100× to 50× greatly enhances the depth of focus from 1.3 μm to 2.5 μm, leading to the focus stability during the operation. The enlargement of viewport in lid insert from 12 mm to 17 mm procures large span of ROI. Lastly, we minimized the image trembling and vibration during the measurement. The main source of image drifting or trembling originates from the thermal expansion of each part during ramping and cooling processes of ICVDM setup. Therefore, we introduced a ball-bearing based linear rail of the chalcogen tube furnace and aluminum bracket to hoist the tube of the chalcogen furnace to accomodate the movement. This greatly reduces the abrupt image trembling or drifting.

Table. Comparison of ICVDM prototype and Mk. I.

| Aspect | Prototype | Mk. I |
| --- | --- | --- |
| Depth of focus (μm) | 1.3 (100×) | 2.5 (50×) |
| Diameter of viewport (mm) | 12 | 17 |
| Minimizing image trembling or drifting | Linear rail w/o ball-bearing | Ball-bearing based linear rail  Aluminum bracket |


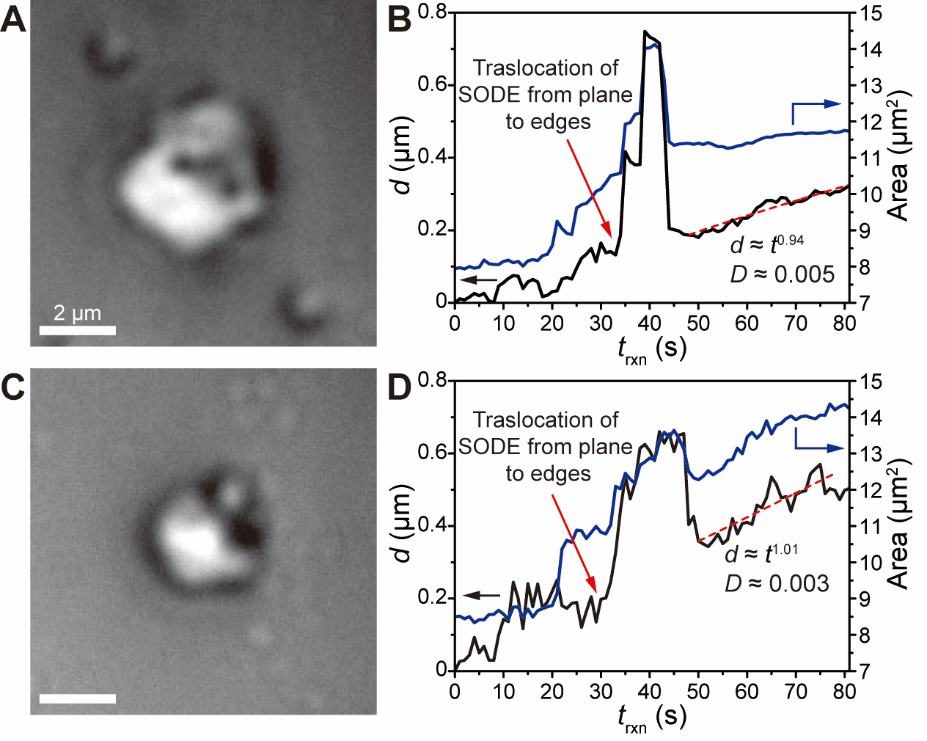


**Figure S1. Additional analysis of directional translational motions of MoS_2_ grains.** (A) Optical image of MoS2 having SODE on top. (B) Analysis of translational motion (left) and area change (right) of the grain. Red dashed line is a linear regression of the translation in (A). (C–D) Similar analysis of different grain.

**
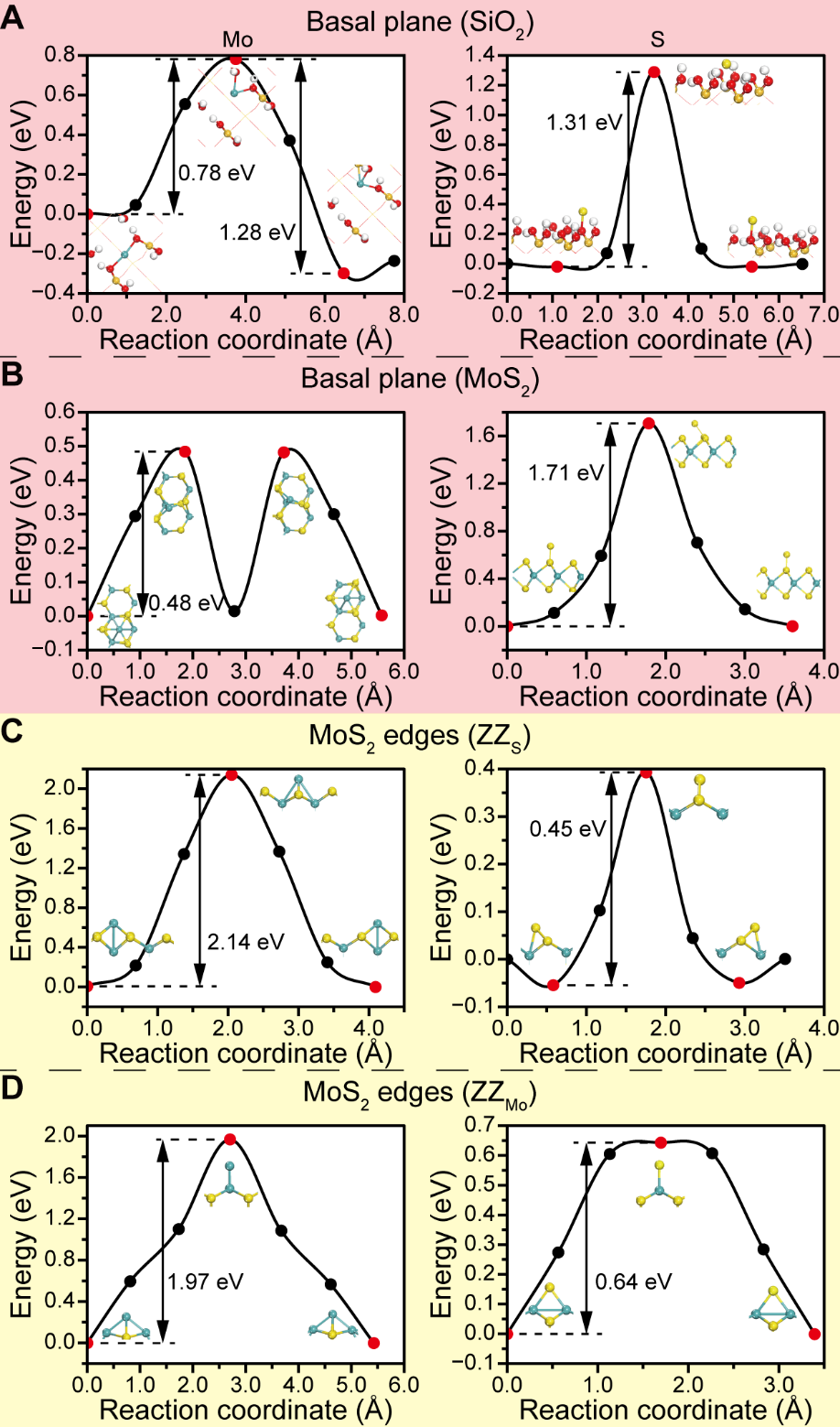
**

**Figure S2.** **Plot of *q_i_* *vs*. reaction coordinate for different substrates.** *q_i_* according to reaction coordinate for (A) SiO_2_ (001) basal plane, (B) MoS_2_ basal plane, (C) ZZ_S_ of MoS_2_, and (D) ZZ_Mo_ of MoS_2_. Lines are drawn for visual aids.

**
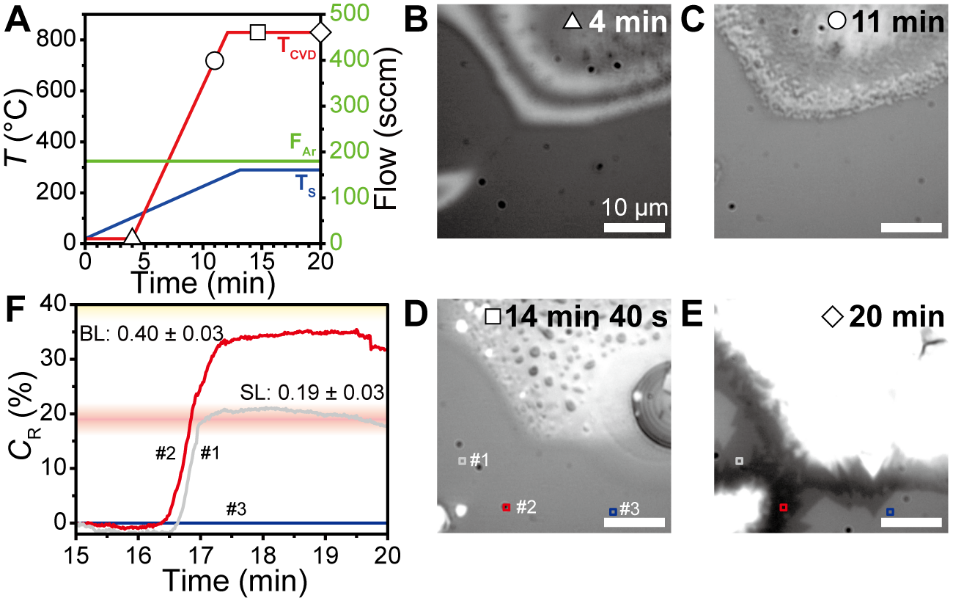
**

**Figure S3.** **Localized MoS_2_ growth without SC.** (A) Plot showing changes in parameters (CVD temperature *T*_CVD_, sulfur temperature *T*_S_, and argon flow *F*_Ar_) over time. (B–E) Time-lapse optical images of the drop-cast MoO_3_ as sulfur gas flows along argon carrier gas. (F) *C*_R_ at each spot indicated in (D–E).

**
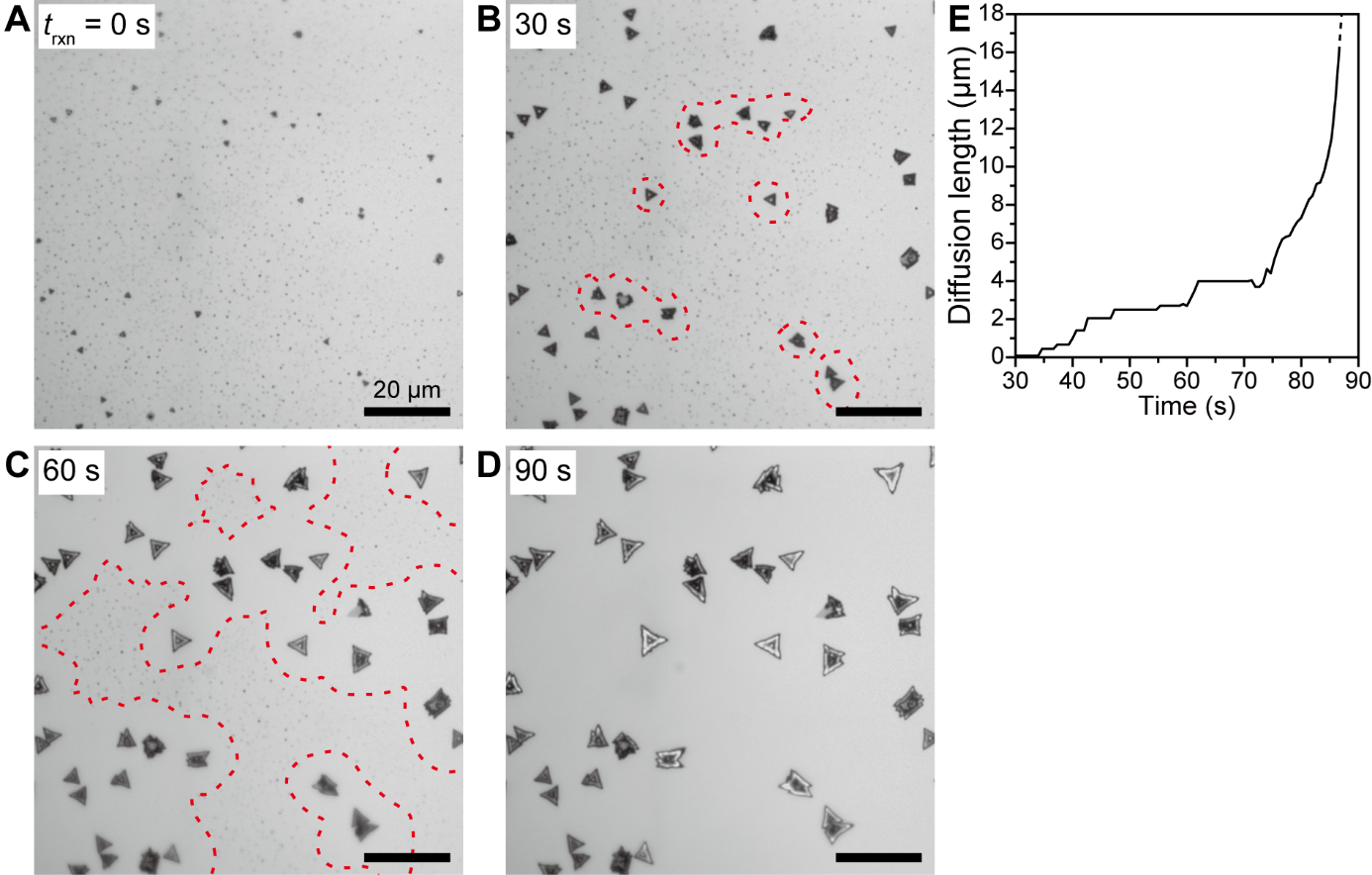
**

**Figure S4.** **2D SODE diffusion promoting MoS_2_ growth.** (A–D) Time-lapse optical images showing the growth of multiple MoS_2_ grain driven by the diffusion of SODE. The red dashed enclosure indicates the area devoid of SODE due to diffusion. Note that SODE diffuses from areas near the MoS_2_ grain, promoting their growth. (E) Plot of the length of SODE-absent regions from MoS_2_ grain as a function of *t*_rxn_.

**
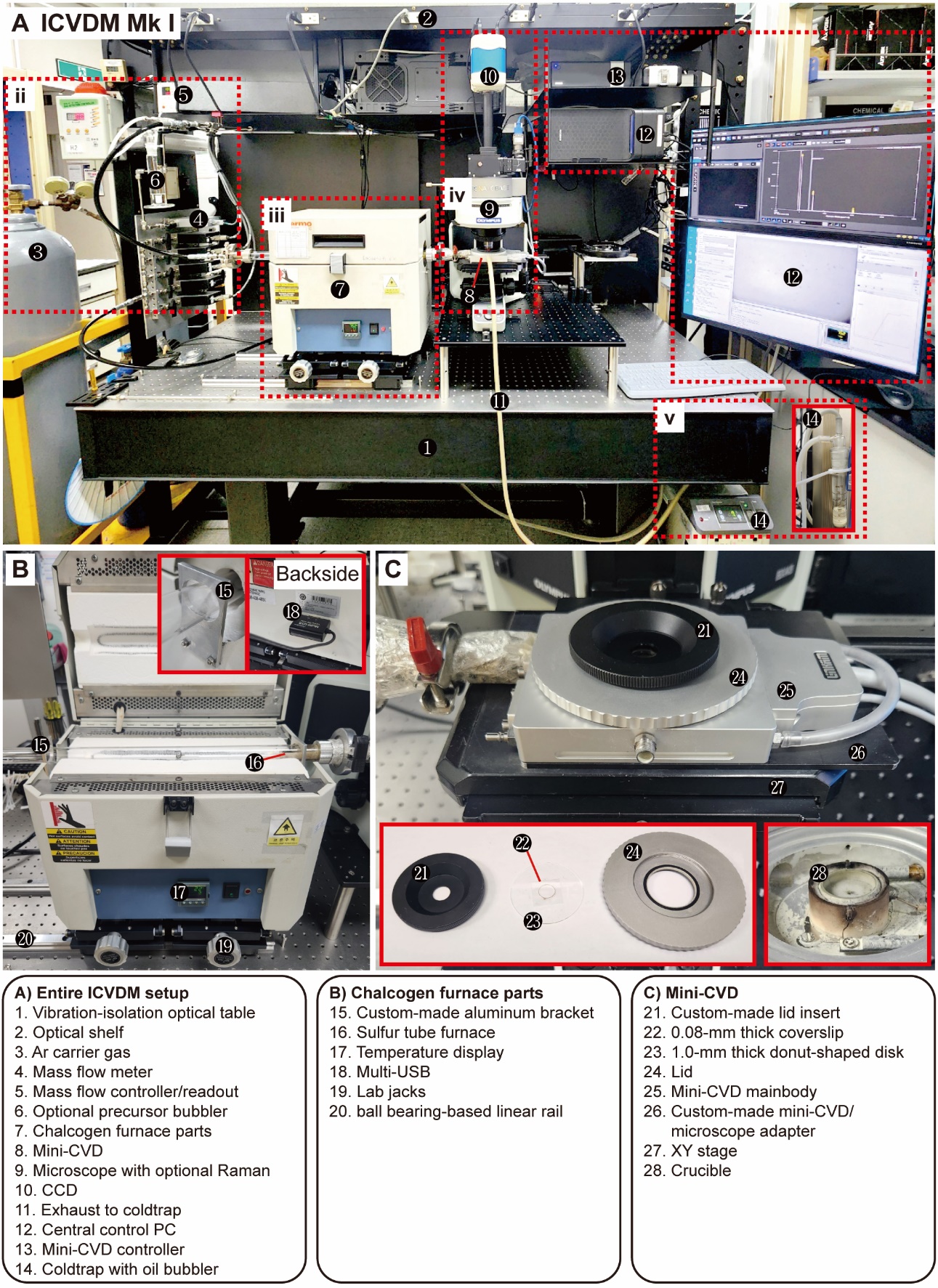
**

**Figure S5. Photographs and details of ICVDM Mk. I.** (A) Photographs of the entire ICVDM Mk. I setup housed by an vibration-isolation optical table and an optical shelf. The dashed boxes denoted by ii), iii), iv), and v) are explained in Note S3. Inset: second oil bubbler. (B) Photographs of chalcogen furnace parts. Insets: custom-made aluminum bracket and multi-USB port. (C) Photographs of mini-CVD parts. Insets: (left) lid assembly consisting of custom-made lid insert, sandwitched donut-shaped quartz disk/0.08-mm thick coverslip and (right) crucible.


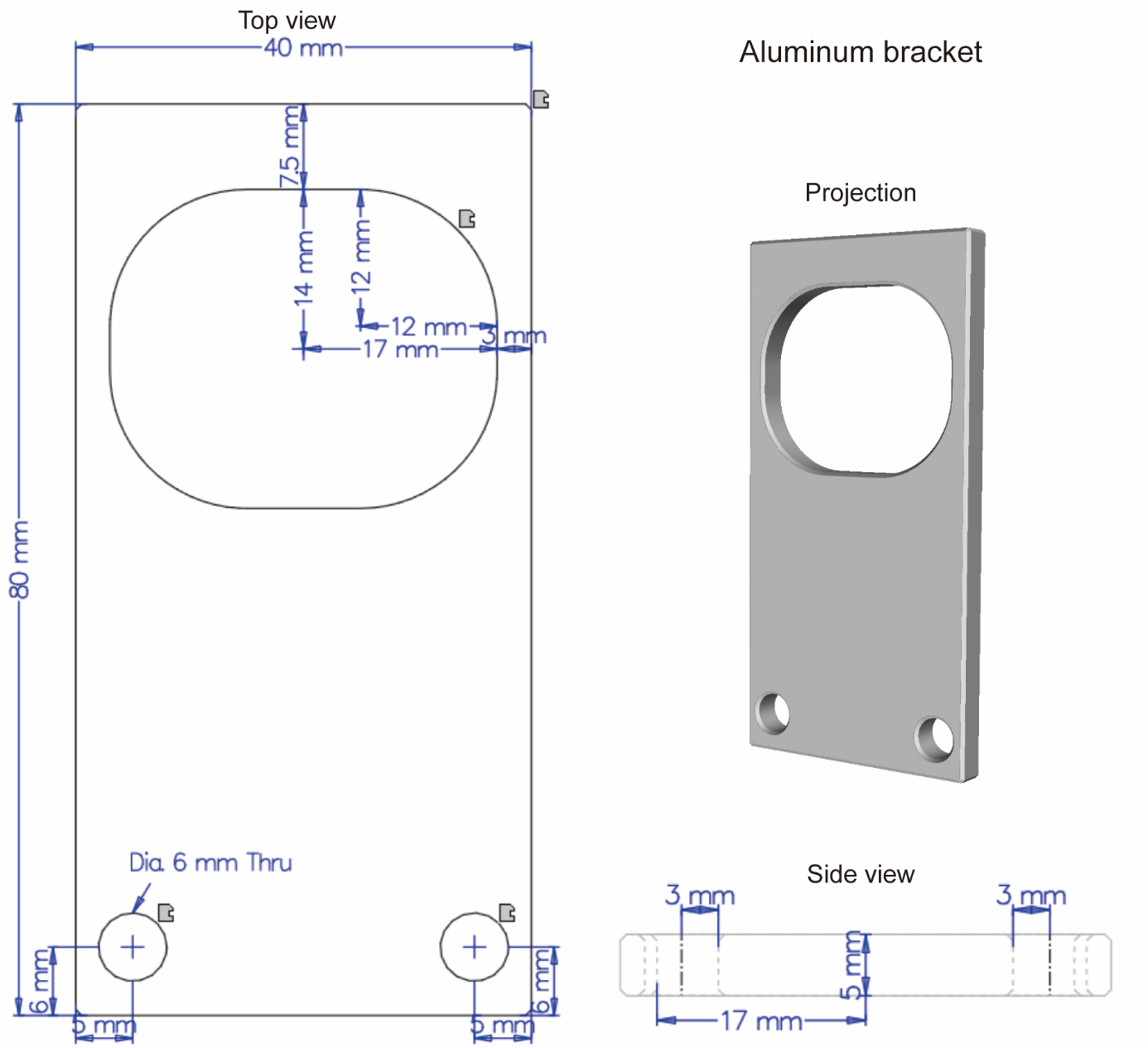


**Figure S6. Drawing of custom-made aluminum bracket.** Top view, side view, and projection of custome-made aluminum bracket.


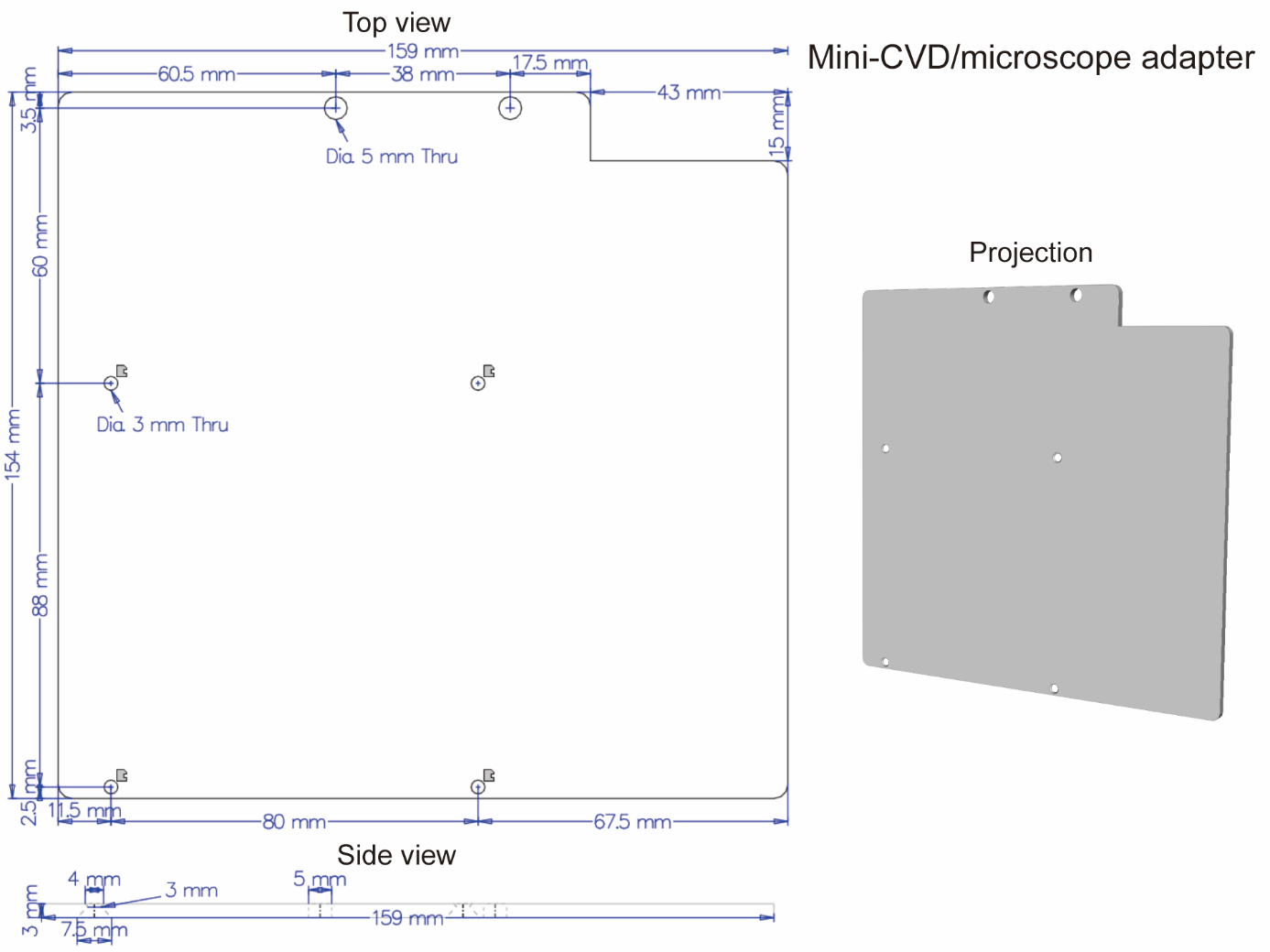


**Figure S7. Drawing of custom-made mini-CVD/XY stage adapter.** Top view, side view, and projection of custome-made mini-CVD/XY stage adapter.


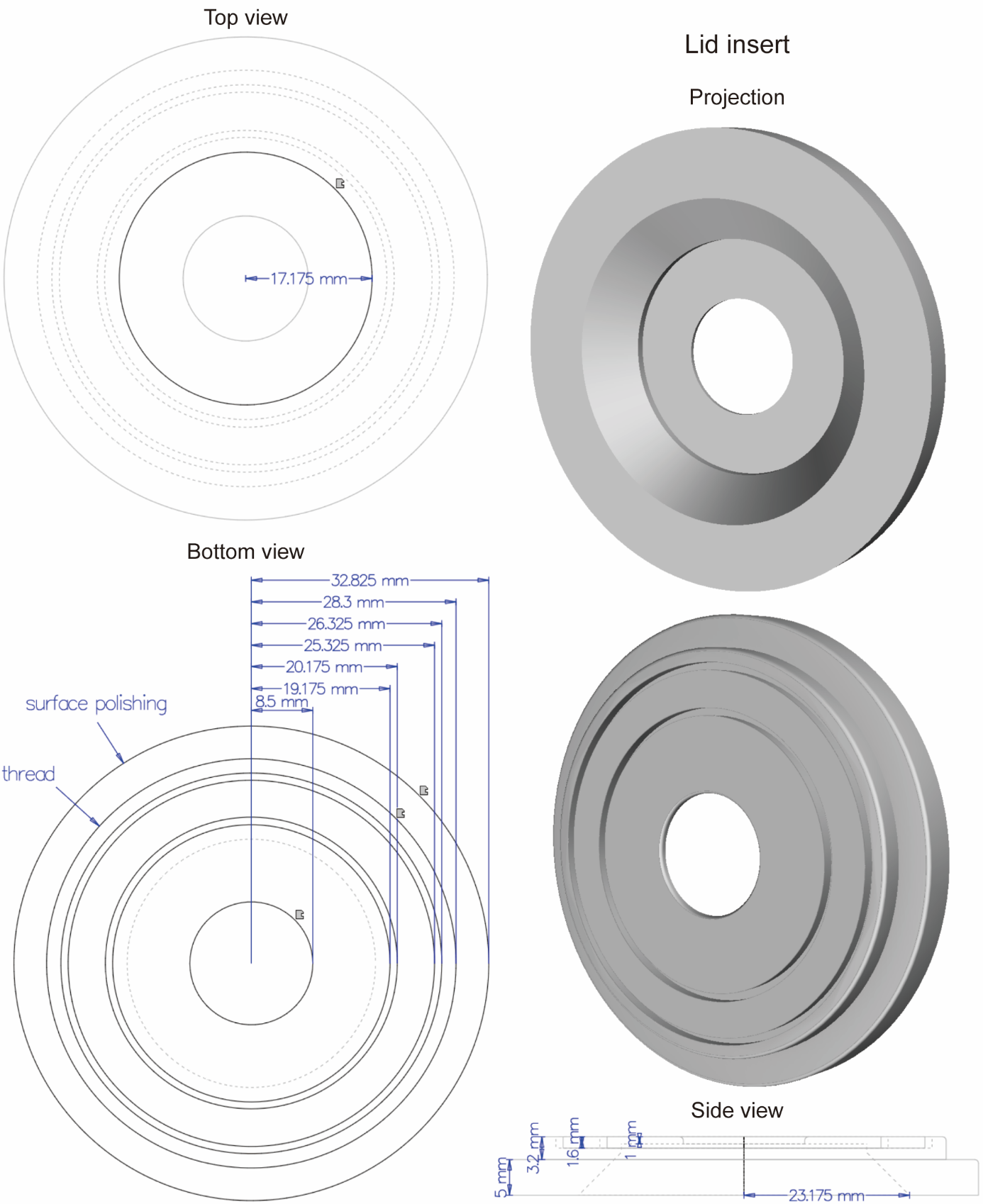


**Figure S8. Drawing of custom-made lid insert.** Top view, bottom view, side view, and projection of custom-made lid insert.


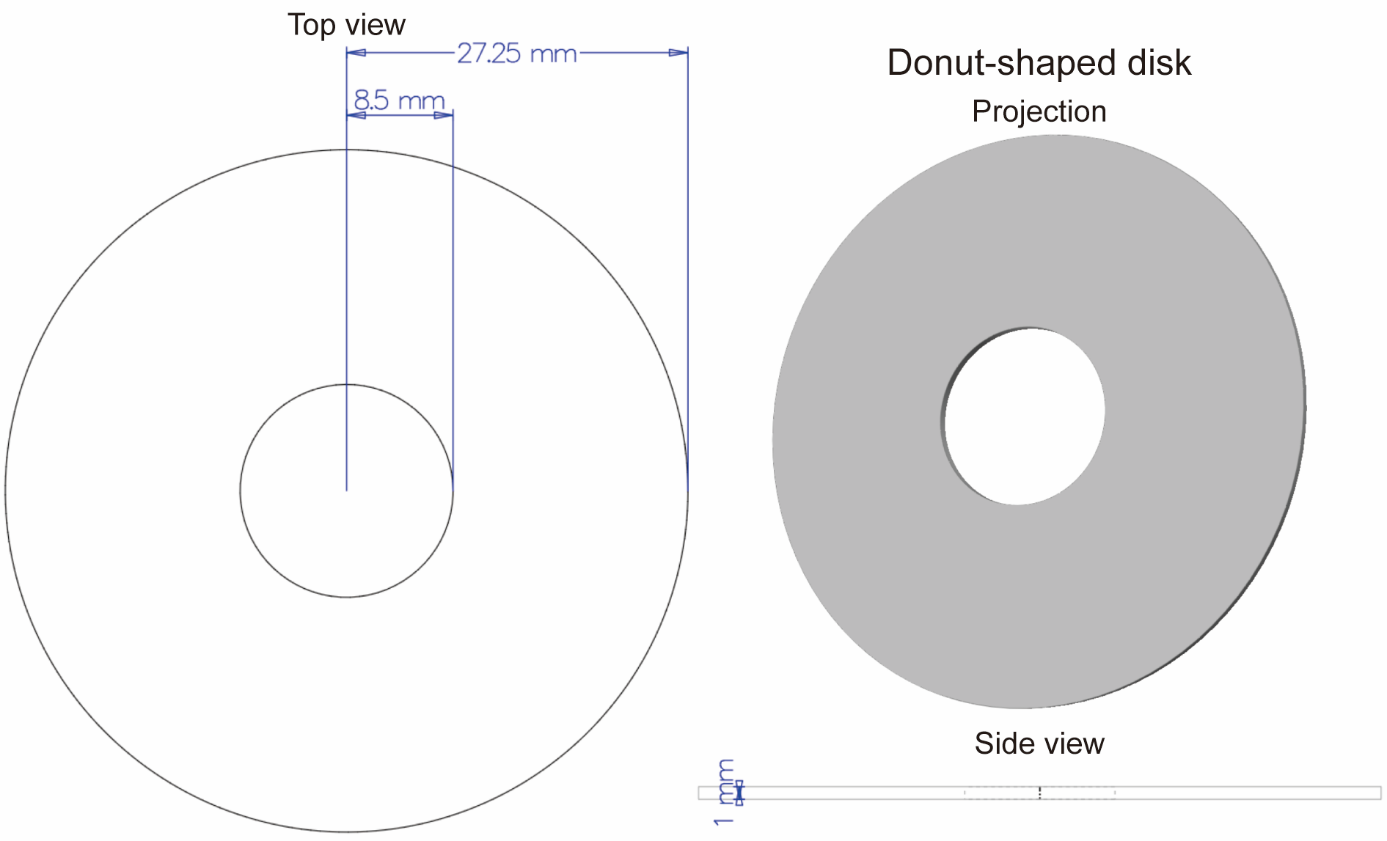


**Figure S9. Drawing of custom-made donut-shaped quartz disk.** Top view, side view, and projection of custom-made donut-shaped quartz disk.

**Table S1.** **Growth kinetics containing *v*_0_, *τ*, and *φ* shown in Figure 2G.**

| Parameters | Bulk I | Bulk II | *t*_rxn_ range for the isolated | | Stepped-on | Overpassed |
| --- | --- | --- | --- | --- | --- | --- |
|  |  |  | 15-50 | 50-65 |  |  |
| *v*_0_ (μm/s) | 0.93 | 0.62 | 0.14 | 0.48 | 0.85 | 0.34 |
| *τ* (s) | 49.02 | 78.44 | 51.08 | 36.40 | 34.97 | 16.67 |
| *φ* (s) | 14.54 | 18.47 | 10.75 | 40.71 | 50.27 | 51.74 |

**Svideo 1.** **A movie clip of translations and rotations of growing triangular MoS_2_ promoted by SODE.** Conditions: 10 frames per second (fps), 830 °C, 100× magnification.

**Svideo 2. A movie clip of substrate-dependent diffusion-mediated growth of MoS_2_.** Conditions: 10 fps, 830 °C, 100× magnification.

**Svideo 3. A movie clip of MoS_2_ grown on a large SODE.** Conditions: 10 fps, 830 °C, 100× magnification.

**Svideo 4. A movie clip of drifting MoS_2_ on mobile SODE.** Conditions: 2 fps, 830 °C, 100× magnification.

**Svideo 5. A movie clip of growing MoS_2_ on growing and merging SODE.** Conditions: 10 fps, 830 °C, 100× magnification.

**Svideo 6. A movie clip of continuous MoS_2_ growth by stitching and etching individual grains.** Conditions: 10 fps, 830 °C, 100× magnification.

**Svideo 7. A movie clip of MoS_2_ growth promoted by the diffusion of submicrometer-size SODEs across the substrate.** Conditions: 30 fps, 750 °C, 50× magnification.

**Cited References**

[1] Oh, J.; Park, M.; Kang, Y.; Ju, S.-Y., Real-Time Observation for MoS_2_ Growth Kinetics and Mechanism Promoted by the Na Droplet, **2024,** *ACS Nano*, 18, 19314-19323, 10.1021/acsnano.4c05586

[2] Nie, Y.; Liang, C.; Zhang, K.; Zhao, R.; Eichfeld, S. M.; Cha, P.-R.; Colombo, L.; Robinson, J. A.; Wallace, R. M.; Cho, K., First Principles Kinetic Monte Carlo Study on the Growth Patterns of WSe_2_ Monolayer, **2016,** *2D Mater.*, 3, 025029, 10.1088/2053-1583/3/2/025029

[3] Kang, Y.; Chang, R.; Ju, S.-Y., Pressure-Dependent Shape and Edge Configurations of MoS_2_ by Kinetic Monte Carlo Simulation, **2024,** *ACS Nano*, 18, 31495-31505, 10.1021/acsnano.4c12342

[4] Rajan, A. G.; Warner, J. H.; Blankschtein, D.; Strano, M. S., Generalized Mechanistic Model for The Chemical Vapor Deposition of 2D Transition Metal Dichalcogenide Monolayers, **2016,** *ACS Nano*, 10, 4330-4344, 10.1021/acsnano.5b07916

[5] Somphonsane, R.; Chiawchan, T.; Bootsa-ard, W.; Ramamoorthy, H., CVD Synthesis of MoS_2_ Using a Direct MoO_2_ Precursor: A Study on the Effects of Growth Temperature on Precursor Diffusion and Morphology Evolutions, **2023,** *Materials*, 16, 4817, 10.3390/ma16134817

[6] Dobkin, D. M.; Zuraw, M. K., *Principles of Chemical Vapor Deposition*. Springer Science & Business Media: **2003**.

[7] Battaile, C. C., The Kinetic Monte Carlo Method: Foundation, Implementation, and Application, **2008,** *Comput. Methods Appl. Mech. Engrg.*, 197, 3386-3398, 10.1016/j.cma.2008.03.010

[8] Markov, I. V., *Crystal Growth for Beginners*. 3rd ed.; World Scientific: **2017**.

[9] Nie, Y.; Liang, C.; Cha, P.-R.; Colombo, L.; Wallace, R. M.; Cho, K., A Kinetic Monte Carlo Simulation Method of van der Waals Epitaxy for Atomistic Nucleation-Growth Processes of Transition Metal Dichalcogenides, **2017,** *Sci. Rep.*, 7, 2977, 10.1038/s41598-017-02919-2

[10] Koo, E.; Ju, S.-Y., Role of Residual Polymer on Chemical Vapor Grown Graphene by Raman Spectroscopy, **2015,** *Carbon*, 86, 318-324, 10.1016/j.carbon.2015.01.055

[11] Koo, E.; Lee, Y.; Song, Y.; Park, M.; Ju, S.-Y., Growth Order-Dependent Strain Variations of Lateral Transition Metal Dichalcogenide Heterostructures, **2019,** *ACS Appl. Electron. Mater.*, 1, 113-121, 10.1021/acsaelm.8b00051

[12] Scientific, L. <https://www.linkam.co.uk/user-guides> (accessed 2025.06.07).
